# Beyond 10-Year Risk: A Cost-Effectiveness Analysis of Statins for the Primary Prevention of Cardiovascular Disease

**DOI:** 10.1161/CIRCULATIONAHA.121.057631

**Published:** 2022-03-07

**Authors:** Ciaran N. Kohli-Lynch, James Lewsey, Kathleen A. Boyd, Dustin D. French, Neil Jordan, Andrew E. Moran, Naveed Sattar, David Preiss, Andrew H. Briggs

**Affiliations:** 1 Center for Health Services and Outcomes Research (C.N.K.-L., D.D.F., N.J.), Feinberg School of Medicine, Northwestern University, Chicago, IL.; 2 Departments of Ophthalmology and Medical Social Science (D.D.F.), Feinberg School of Medicine, Northwestern University, Chicago, IL.; 3 Psychiatry & Behavioral Sciences and Preventive Medicine (N.J.), Feinberg School of Medicine, Northwestern University, Chicago, IL.; 4 Health Economics and Health Technology Assessment (C.N.K.-L., J.L., K.A.B., A.H.B.), University of Glasgow, UK.; 5 Institute of Cardiovascular and Medical Sciences (N.S.), University of Glasgow, UK.; 6 Center of Innovation for Complex Chronic Healthcare, Hines VA Hospital, Chicago, IL (D.D.F., N.J.).; 7 Division of General Medicine, Columbia University Irving Medical Center, New York (A.E.M.).; 8 Medical Research Council Population Health Research Unit, Clinical Trial Service Unit, and Epidemiological Studies Unit, Nuffield Department of Population Health, University of Oxford, UK (D.P.).; 9 Department of Health Services Research and Policy, London School of Hygiene and Tropical Medicine, UK (A.H.B.).

**Keywords:** cardiovascular diseases, cholesterol, hydroxymethylglutaryl-CoA reductase, risk

## Abstract

Cholesterol guidelines typically prioritize primary prevention statin therapy on the basis of 10-year risk of cardiovascular disease. The advent of generic pricing may justify expansion of statin eligibility. Moreover, 10-year risk may not be the optimal approach for statin prioritization. We estimated the cost-effectiveness of expanding preventive statin eligibility and evaluated novel approaches to prioritization from a Scottish health sector perspective.

**Methods:**

A computer simulation model predicted long-term health and cost outcomes in Scottish adults ≥40 years of age. Epidemiologic analysis was completed using the Scottish Heart Health Extended Cohort, Scottish Morbidity Records, and National Records of Scotland. A simulation cohort was constructed with data from the Scottish Health Survey 2011 and contemporary population estimates. Treatment and cost inputs were derived from published literature and health service cost data. The main outcome measure was the lifetime incremental cost-effectiveness ratio, evaluated as cost (2020 GBP) per quality-adjusted life-year (QALY) gained. Three approaches to statin prioritization were analyzed: 10-year risk scoring using the ASSIGN score, age-stratified risk thresholds to increase treatment rates in younger individuals, and absolute risk reduction (ARR)–guided therapy to increase treatment rates in individuals with elevated cholesterol levels. For each approach, 2 policies were considered: treating the same number of individuals as those with an ASSIGN score ≥20% (age-stratified risk threshold 20, ARR 20) and treating the same number of individuals as those with an ASSIGN score ≥10% (age-stratified risk threshold 10, ARR 10).

**Results:**

Compared with an ASSIGN score ≥20%, reducing the risk threshold for statin initiation to 10% expanded eligibility from 804 000 (32% of adults ≥40 years of age without CVD) to 1 445 500 individuals (58%). This policy would be cost-effective (incremental cost-effectiveness ratio, £12 300/QALY [95% CI, £7690/QALY–£26 500/QALY]). Incremental to an ASSIGN score ≥20%, ARR 20 produced ≈8800 QALYs and was cost-effective (£7050/QALY [95% CI, £4560/QALY–£10 700/QALY]). Incremental to an ASSIGN score ≥10%, ARR 10 produced ≈7950 QALYs and was cost-effective (£11 700/QALY [95% CI, £9250/QALY–£16 900/QALY]). Both age-stratified risk threshold strategies were dominated (ie, more expensive and less effective than alternative treatment strategies).

**Conclusions:**

Generic pricing has rendered preventive statin therapy cost-effective for many adults. ARR–guided therapy is more effective than 10-year risk scoring and is cost-effective.

**Clinical Perspective**

**What Is New?**

- The advent of generic pricing has rendered preventive statin therapy cost-effective for many adults.
- Absolute risk reduction–guided statin therapy on the basis of 10-year cardiovascular disease risk and non–high-density lipoprotein cholesterol levels is cost-effective and would improve population health.
- Age-stratified risk thresholds were more expensive and less effective than alternative approaches to statin prioritization.

**What Are the Clinical Implications?**

- Guideline committees should expand statin eligibility and consider new ways to allocate statins on the basis of absolute risk reduction rather than 10-year risk thresholds.
- The optimal prevalence of statin eligibility is sensitive to patient preference for daily pill-taking.

Cardiovascular disease (CVD) is the leading cause of morbidity and mortality worldwide.^1^ In the United Kingdom, >140 000 deaths were attributable to CVD in 2018 and rates are disproportionately high in Scotland.^2,3^ Hydroxymethylglutaryl–coenzyme A reductase inhibitors (statins) are a cornerstone treatment for the primary prevention of CVD. They are a first-line treatment for lipid-lowering therapy, their efficacy and safety has been established in high-quality clinical trials, and recent price reductions have made this drug class affordable.

Individuals without CVD are often prioritized for preventive statin therapy on the basis of 10-year risk of experiencing a primary CVD event, estimated using 10-year CVD risk scores.^4–7^ The National Institute for Health and Care Excellence in England and Wales recommends statins for individuals 40 years of age and older with a 10-year CVD risk score ≥10%, type 1 diabetes, chronic kidney disease, or total cholesterol ≥7.5 mmol/L.^5,8^ The risk threshold for statin eligibility had previously been 20%.

Unlike the National Institute for Health and Care Excellence, the Scottish Intercollegiate Guidelines Network has retained a risk threshold of 20% for statin eligibility.^6^ The Scottish Intercollegiate Guidelines Network recommends that risk be assessed using the ASSIGN risk score, which was developed with Scottish data. The risk factors included in the ASSIGN score are age, sex, diabetes, systolic blood pressure, total cholesterol (TC) level, high-density lipoprotein cholesterol (HDL-C) level, cigarettes per day, Scottish Index of Multiple Deprivation, and family history of CVD.

Reducing the risk threshold in England and Wales was partly justified by the advent of generic pricing for most statin formulations. Generic statin pricing expanded the proportion of the population who would be cost-effective to treat.^9–12^ The cost-effectiveness of lowering the threshold or pursuing alternative prioritization strategies in Scotland has not been evaluated. Most other countries still use 10-year risk thresholds.^13^

We aimed to estimate the cost-effectiveness of lowering the 10-year CVD risk threshold for statin eligibility in Scotland and estimate the cost-effectiveness of novel approaches to statin prioritization, an analysis highly relevant to future statin prescribing in many countries worldwide.

## Methods

The Scottish CVD Policy Model, the decision-analytic model used in our analysis, and the program code used to run the model are publicly available and can be accessed at https://github.com/yiqiaoxin/CVDmodel. The Scottish Health Survey is publicly available from the UK Data Service and can be accessed at https://beta.ukdataservice.ac.uk/datacatalogue/series/series?id=2000047.

### Novel Approaches to Statin Prioritization

Novel approaches to statin prioritization may be preferable to 10-year risk scoring. Two alternative approaches to statin prioritization are age-stratified risk thresholds and absolute risk reduction (ARR).

### Age-Stratified Risk Thresholds

The age-stratified risk threshold approach involves setting separate risk thresholds for statin initiation in different age groups. This approach has been introduced in Norway and is predicated on the concept that risk scores are poorly calibrated for some subsets of the population without CVD.^14^ This includes younger individuals who are at high risk of developing CVD relative to their age group peers.^15^

Some CVD risk factors are generic to a range of adverse health conditions. For example, age is a risk factor for CVD but is also predictive of non-CVD mortality. Ten-year CVD risk scores disregard the competing risk of non-CVD mortality and therefore may overstate potential benefit from preventive therapy. Using age-stratified risk thresholds to target treatment at younger individuals with unhealthy levels of modifiable risk factors may produce greater health benefits than current practice.

### Absolute Risk Reduction

The ARR approach to statin prioritization recognizes that reduction in cholesterol is the major driver of statin benefit and that this can be estimated from baseline cholesterol level.^16^ Several major clinical trials have analyzed the effect of statins on CVD risk. This enables powerful inference of statin effectiveness in patient subgroups. A key finding has been that relative risk reduction (RRR) of CVD from statin therapy is near constant at ≈22% per 1.0 mmol/L reduction in low-density lipoprotein cholesterol (LDL-C) level over 5 years.^17^ Furthermore, statins tend to produce a greater reduction in individuals with higher baseline LDL-C level. Combining these 2 findings—RRR applied to baseline absolute risk—suggests that individuals with higher baseline LDL-C level achieve greater ARR from statin therapy.

Consider 2 individuals with the same ASSIGN 10-year risk score: 1 with an LDL-C level of 4.0 mmol/L and 1 with an LDL-C level of 2.0 mmol/L. If atorvastatin 20 mg reduces LDL-C level by ≈40%, its use in these individuals yields reductions of 1.6 mmol/L and 0.8 mmol/L, respectively. This translates to a 33% RRR for the former individual and an 18% RRR for the latter.

To prevent CVD events, we are ultimately concerned with ARR. Thanassoulis et al^16^ developed an equation to predict 10-year ARR from statin therapy. This equation predicts that ARR from statins is the product of an individual’s baseline 10-year risk and the baseline LDL-C level ($ldl_{b}$). The equation is as follows:


$$\text{ARR}=S_{un}{}^{x}-S_{un};\;x=HR^{ldl_{b}\text{*}40\text{\%}}.$$ (1)

$S_{un}$ and HR represent 10-year untreated survival and the hazard ratio associated with a unitary reduction in LDL-C level, respectively. The equation assumes that statins produce a 40% reduction in LDL-C level. We modified this equation to account for the effect of statins on non–HDL-C rather than LDL-C level in our analysis.

### Scottish CVD Policy Model

The Scottish CVD Policy Model was used to estimate the cost-effectiveness of different statin policies (Tables S1–S6). This open-source, decision-analytic model was developed in the *R* programming language (version 4.0.4; R Core Team)^18^ and has been validated in the Scottish population (Figures S1 and S2).^19–21^ The model predicts life expectancy, quality-adjusted life-years (QALYs), and health care costs for individuals receiving care in the Scottish National Health Service on the basis of their ASSIGN risk factors.

**Figure 1.**
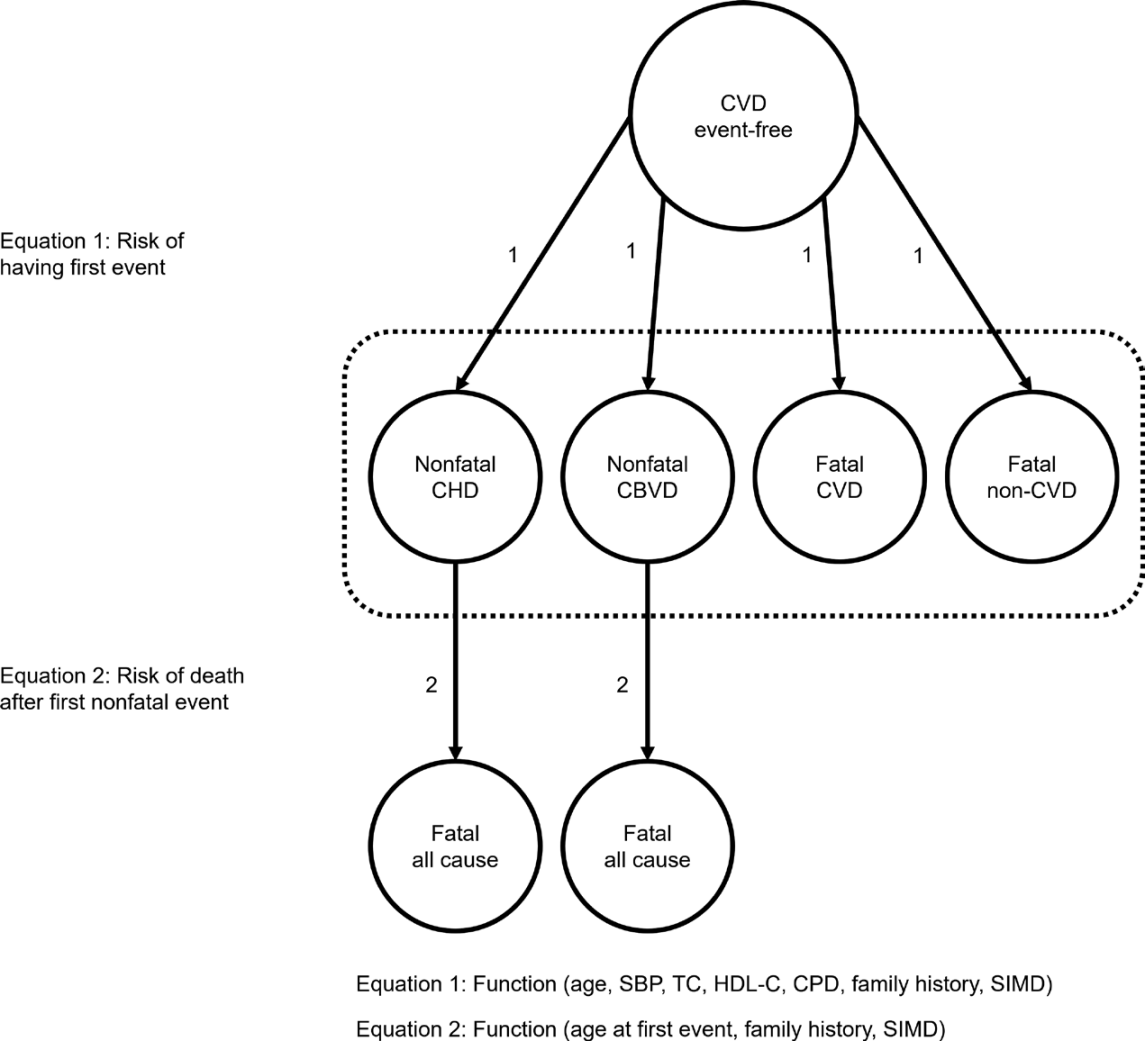
**Structure of the Scottish Cerebrovascular Disease Policy Model.** CBVD indicates cerebrovascular disease; CHD, coronary heart disease; CPD, cigarettes per day; CVD, cardiovascular disease; HDL-C, high-density lipoprotein cholesterol; SBP, systolic blood pressure; SIMD, Scottish Index of Multiple Deprivation; and TC, total cholesterol.

Figure 1 shows a diagram of the model. Individuals enter CVD-free and transition to 1 of 4 primary event types throughout their lives: nonfatal coronary heart disease (CHD), nonfatal cerebrovascular disease, fatal CVD, or fatal non-CVD. After the occurrence of a nonfatal primary event, individuals progress to an absorbing state representing all-cause mortality.

Probability of state transition was determined by competing risk parametric survival analysis of a linked Scottish Heart Health Extended Cohort–Scottish Morbidity Records data set.^22,23^ The Scottish Heart Health Extended Cohort was used in the construction of the ASSIGN risk score. Researchers collected baseline risk factor information for >13 000 Scottish adults commencing in 1986. The Scottish Morbidity Records is an electronic database that records all hospitalized events that occur in the Scottish National Health Service. Baseline risk factors were linked with the Scottish Morbidity Records through participants’ unique National Health Service identification number.

Each state in the model has an assigned disutility value, derived from a survey of the Scottish population. Individuals who have not experienced a primary CVD event are attributed a background health-related quality of life, disaggregated by age and sex. Individuals inhabiting 1 of the 2 nonfatal chronic CVD states are assigned a decrement to their background quality of life, determined by the type of primary event (CHD or cerebrovascular disease). Within the chronic disease states, individuals may experience further utility decrements attributable to secondary CVD events (ie, myocardial infarction, stroke, transient ischemic attack, heart failure, peripheral artery disease, other CVD event).

All states in the model are assigned health care costs, derived from a combination of Scottish Morbidity Records data and English tariffs for elective and nonelective hospitalizations.^24^ When the primary event is nonfatal, linear equations predict pre- and postevent hospitalization costs. These equations include age at primary event, Scottish Index of Multiple Deprivation, and family history of CVD as covariates.

The model estimates outcomes through cohort simulation. It deterministically assigns individuals to health states on the basis of the Scottish Heart Health Extended Cohort–Scottish Morbidity Records risk functions and a profile of CVD risk factors. Health and cost estimates are produced for each risk factor profile by summing the probability-weighted outcomes associated with each health state that an individual may encounter. This process is outlined in the Supplemental Material. Heterogeneity in the population is reflected by simulating multiple risk factor profiles that are reflective of the CVD-free Scottish population.

### Simulation Data

To simulate the Scottish CVD-free population, information on risk factor and age distributions was required. Our analysis was completed using a combination of the Scottish Health Survey (SHS) 2011 and contemporary population estimates from the National Records of Scotland.^25,26^

The SHS is a study of public health and ASSIGN risk factor values can be derived for all respondents from the 2011 survey data. The SHS 2011 is the most contemporaneous data set regarding the distribution of CVD risk factors in Scotland. Subsequent waves of the survey have not included a nurse visit so have not collected blood samples or recorded systolic blood pressure.

We excluded individuals <40 years of age and those with existing CVD from our data set. Individuals currently receiving statins were “detreated” by modifying cholesterol levels according to treatment effects observed in randomized clinical trials.^17^ A relatively small number of respondents received nurse visits. This meant that data were sparse for 3 important covariates: TC level, HDL-C level, and systolic blood pressure. For most individuals, we assumed these variables were missing at random and they were imputed with stochastic regression.^27^ For individuals who refused nurse visits, we performed multiple imputation (Table S7).^28^ Individuals with familial hypercholesterolemia, defined as TC level ≥7.5 mmol/L and a family history of premature CVD or TC level ≥8.0 mmol/L, are high priority for statin therapy and were omitted from the analysis.^6^ In a scenario analysis, we further excluded individuals with diabetes.

More information on the SHS and our imputation procedure is included in the Supplemental Material.

### Treatment Criteria

We first aimed to establish the cost-effectiveness of expanding statin eligibility. This was achieved by analyzing 3 treatment strategies in the SHS cohort: no treatment, statins for individuals with an ASSIGN score ≥20% (ASSIGN 20), and statins for individuals with an ASSIGN score ≥10% (ASSIGN 10).

Our second objective was to estimate the cost-effectiveness of novel approaches to statin prioritization. Age-stratified risk thresholds and ARR were analyzed. Two strategies were defined for each novel strategy: 1 treating approximately the same number of people as ASSIGN 20 (age-stratified risk threshold 20, ARR 20) and 1 treating approximately the same number of people as ASSIGN 10 (age-stratified risk threshold 10, ARR 10).

For age-stratified risk threshold policies, we set separate risk thresholds for 5-year age groups from ages 40 to 79 years and individuals ≥80 years of age. These thresholds targeted treatment at individuals who were at high risk relative to their age group peers, thus increasing the proportion of younger individuals who were treated (Supplemental Material).

We estimated ARR from statins for everyone in the SHS cohort using a modified form of Equation 1. Because of data limitations, LDL-C level was replaced with non–HDL-C level. The percentage reduction in non–HDL-C level and hazard ratio per 1.0 mmol/L reduction were altered in the equation accordingly. We established ARR thresholds that would treat the same proportion of the population as ASSIGN 20 and ASSIGN 10, respectively.

The specific age-stratified risk threshold and ARR policies that we analyzed are shown in Table 1. The proportions of the Scottish population eligible for treatment under different risk and ARR thresholds are presented in Table 1 and Figures S3 through S5. Ten-year risk, age-stratified risk threshold, and ARR strategies that treated the same number of people were compared using traditional cost-effectiveness decision rules.^29^

**Table 1.**
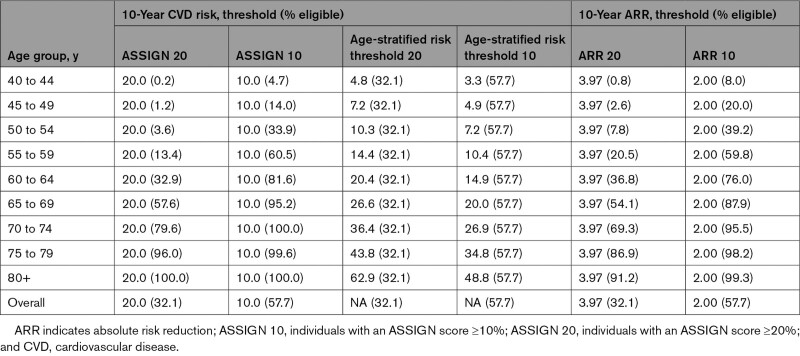
Statin Eligibility Criteria and Percentage Eligible for Treatment Under Different Treatment Strategies

### Statin Treatment Measures

Statins reduced risk of nonfatal CHD, nonfatal cerebrovascular disease, and fatal CVD in the model. This was achieved by lowering individuals’ non–HDL-C levels (Table 2). Patients receiving statins incurred side effects and accumulated treatment and monitoring costs (Table S8).

**Table 2.**
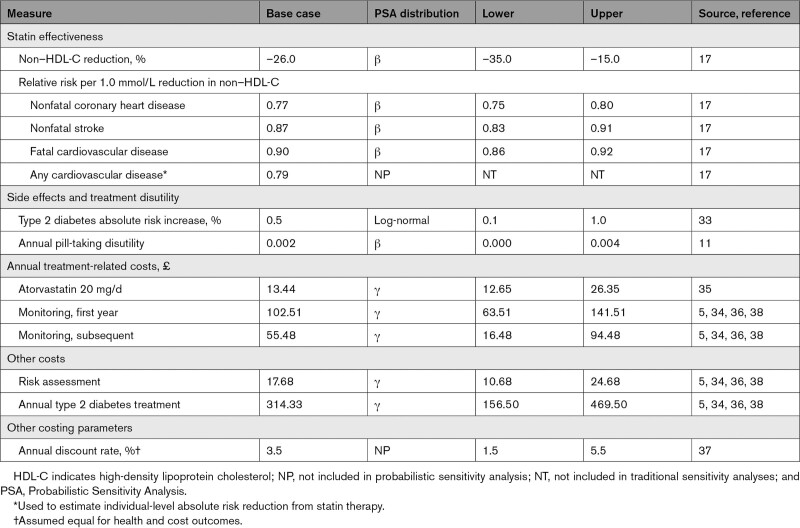
Intermediate-Intensity Statin Treatment Measures

The Scottish CVD Policy Model does not include LDL-C level as a predictor of CVD risk. Instead, it includes TC level and HDL-C level. The SHS only collected data on TC level and HDL-C level. Evidence suggests that statins produce a 26% reduction in non–HDL-C level and are associated with relative risks of 0.77, 0.87, and 0.90 for nonfatal CHD, nonfatal stroke, and fatal CVD per 1.0 mmol/L reduction in non–HDL-C level, respectively.^17^ These values were derived from secondary analysis of a Cholesterol Treatment Trialists’ meta-analysis and are likely conservative estimates of the effect of statins on CVD risk as mediated by non–HDL-C level reduction.^30^

Statins are a relatively safe treatment with a well-established side effect profile.^31,32^ In the model, statins increased absolute risk of new onset diabetes by 0.5%.^33^ An annual pill-taking disutility of 0.002 QALYs was also applied.^11^

Statin costs were obtained from the British National Formulary.^34^ An annual cost of £13.44 was applied for every year on statin therapy, representing the annual National Health Service drug tariff price for generic atorvastatin 20 mg.^35^ Alternative moderate-intensity statins are available, and their prices were used to define upper and lower limits in sensitivity analyses.

All individuals experienced screening costs on entering the model. Patients prescribed statins were assigned monitoring costs, which were largely obtained from a cost-effectiveness analysis of statin policy conducted for the National Institute for Health and Care Excellence and an analysis of unit costs for health and social care in England and Wales.^5,36^ Additional costs were added for each statin user attributable to the increased risk of diabetes.

### Statistical Analysis

The Scottish CVD Policy Model simulated the cost-effectiveness of different risk- and ARR-guided statin policies. A baseline simulation predicted treatment-free health and cost outcomes. Next, model measures were altered to simulate the benefits, side effects, and costs associated with moderate-intensity statin therapy. If individuals in the SHS cohort met treatment criteria, they received the benefits and costs associated with statin therapy. Otherwise, they incurred no treatment costs or benefits. One iteration of the model involved simulating every individual in the SHS cohort.

This study followed the Consolidated Health Economic Evaluation Reporting Standards reporting guideline (Table S9). A health sector perspective was adopted, which accounted for all screening, statin treatment, monitoring, CVD, and background health care costs incurred by the Scottish National Health Service. The primary outcome considered was the incremental cost (2020 GBP) per QALY gained for different treatment strategies with a lifetime horizon. Intermediate outcomes recorded were primary CVD events prevented, life-years gained, and disaggregated health care costs. Future costs and health benefits were discounted at a rate of 3.5% annually.^37^ Model costs were inflated by 12.5% to account for health services pay and price inflation from 2014 to 2020.^38^ Cost-effectiveness analysis was performed with a health sector perspective and a strategy was deemed cost-effective if its incremental cost-effectiveness ratio (ICER) was <£20 000/QALY.^39,40^ This is a standard threshold used in cost-effectiveness analyses in Scotland and the United Kingdom.^6,37^

Simulation analysis was completed by stochastically sampling Table 2 measure distributions and risk factor hazard ratios, then estimating costs and QALYs for the respective treatment strategies in 1000 independent iterations. Correlation between risk factor hazard ratios was accounted for with Cholesky decomposition.^41^ Base case results were derived from the mean values of the probabilistic analyses and 95% CIs were presented as the 2.5th and 97.5th percentiles of the 1000 iterations. Cost-effectiveness acceptability curves were produced with results from the probabilistic analysis.

Population estimates from the National Records of Scotland were used to project results onto the wider Scottish population.^25^ The number of individuals in each age group was derived from these estimates. This number was multiplied by the respective proportion of CVD-free individuals in each age group in SHS 2011 to obtain the number of individuals eligible for preventive treatment. The outcomes observed in the simulation were projected onto the Scottish population by multiplying average age group–level outcomes in the simulation by the number of eligible patients in the wider population.

### Sensitivity Analyses

Sensitivity analyses assessed the effect of key modeling measures on cost-effectiveness estimates. Table 2 lists the lower and upper values used in these analyses. Results from the sensitivity analysis were synthesized in tornado diagrams. These diagrams showed the effect of modeling assumptions on the cost-effectiveness of a treatment policy, represented by change in net monetary benefit. Unlike the ICER, net monetary benefit is a continuously defined, linear measure of cost-effectiveness and an increase represents increased cost-effectiveness.^42^ We estimated the net monetary benefit for all strategies at a range of values between the lower and upper limits defined in Table 2.

In our base case, we assumed full adherence to statin therapy (ie, effect size equal to effects observed in clinical trials). In a scenario analysis, we assumed 67% of patients would continue treatment in the first, 53% in the second, and 50% in subsequent years of statin initiation.^43^ Treatment efficacy, side effects, and monitoring costs were only experienced by persistent statin users. In a further scenario analysis, we excluded individuals with diabetes from the prospective patient population and assumed that they would be treated regardless of prioritization criteria. We also considered the net monetary benefit of each treatment strategy over a wide range of values for pill-taking disutility to establish optimal treatment strategies dependent on a patient’s aversion to taking pills regularly.

Whereas the primary analysis was limited to 2 ASSIGN score thresholds for statin initiation, we considered the cost-effectiveness of reducing the threshold below ASSIGN 10 in a sensitivity analysis. We considered the incremental cost-effectiveness of reducing the threshold at 1% increments from ASSIGN 20 to treatment of the entire CVD-free adult population.

Institutional review board approval was not required because the study was a secondary analysis of publicly available and deidentified data.

## Results

Descriptive statistics for the final data set and treatment-eligible populations are displayed in Table S10. The overall population was disproportionately female, likely because we excluded individuals with established CVD. Age-stratified risk thresholds and ARR strategies reduced the average age at treatment compared with standard 10-year risk scoring. The ARR strategies treated patients with higher TC level, higher non–HDL-C level, and lower HDL-C level compared with the alternatives.

Our first objective was to estimate the cost-effectiveness of extending preventive statin therapy eligibility (Table 3). ASSIGN 20 was cost-effective compared with no treatment (ICER, £3850/QALY [95% CI, £957/QALY–£6770/QALY]). Implementing ASSIGN 10 would also be cost-effective (ICER vs ASSIGN 20, £12 300/QALY [95% CI, £7690/QALY–£26 500/QALY]).

**Table 3.**
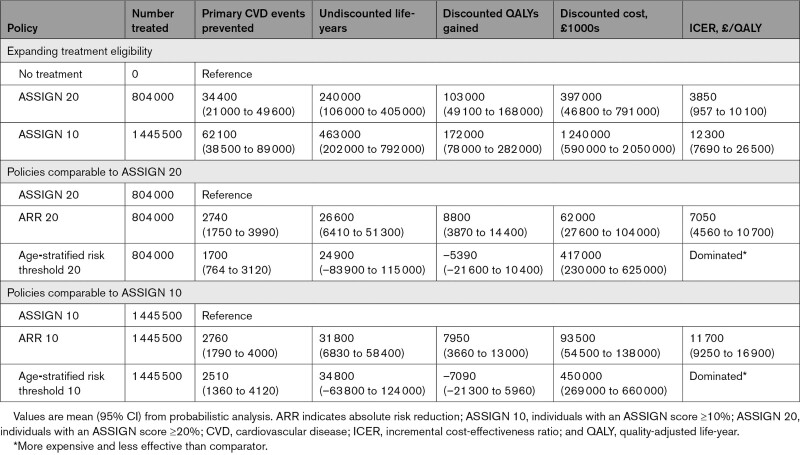
Cost-Effectiveness of Statin Treatment Strategies

Reducing the ASSIGN risk threshold from 20% to 10% would extend statin eligibility by ≈641 500 individuals, from 804 000 to 1 445 500. Statin eligibility would increase from 32% to 58% of the Scottish CVD-free population ≥40 years of age. Reducing the threshold would prevent ≈27 700 primary CVD events and produce ≈223 000 life-years and 69 000 discounted QALYs. Sensitivity analysis showed that it would be cost-effective to further reduce the threshold to ≈8.0% (Table S11).

**Figure 2.**
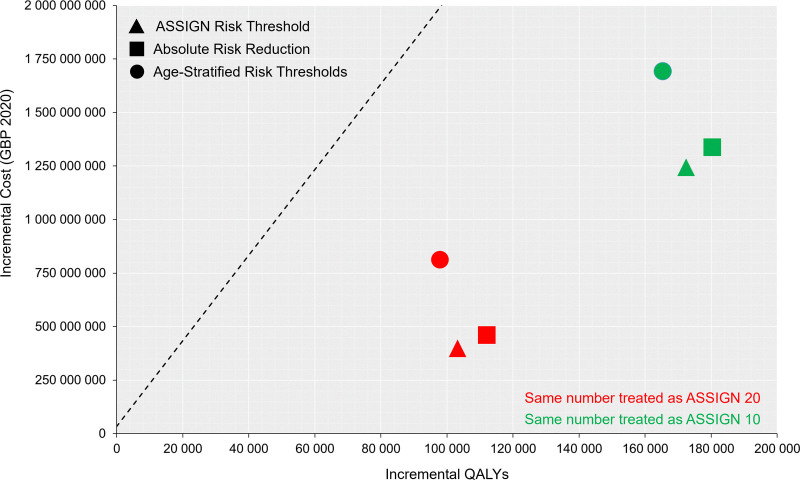
**Cost-effectiveness plane for all treatment strategies.** The dashed line represents cost-effectiveness threshold of £20 000/quality-adjusted life-year (QALY). ASSIGN 10 indicates individuals with an ASSIGN score ≥10%; and ASSIGN 20, individuals with an ASSIGN score ≥20.

Our second objective was to estimate the cost-effectiveness of novel approaches to statin prioritization (Figure 2). Both age-stratified risk policies were dominated, meaning they were more expensive and less effective than alternative treatment options. Incremental to ASSIGN 20, ARR 20 treated approximately the same number of individuals, produced ≈8800 QALYs, and had an ICER of £7050/QALY (95% CI, £4560/QALY–£10 700/QALY). Incremental to ASSIGN 10, ARR 10 treated approximately the same number of individuals, produced ≈7950 QALYs, and had an ICER of £11 700/QALY (95% CI, £9250/QALY–£16 900/QALY).

Compared with no treatment, all statin strategies led to large reductions in CVD-related health care costs (Table S12). However, these were offset by non-CVD health care, statin, monitoring, and risk assessment costs.

**Figure 3.**
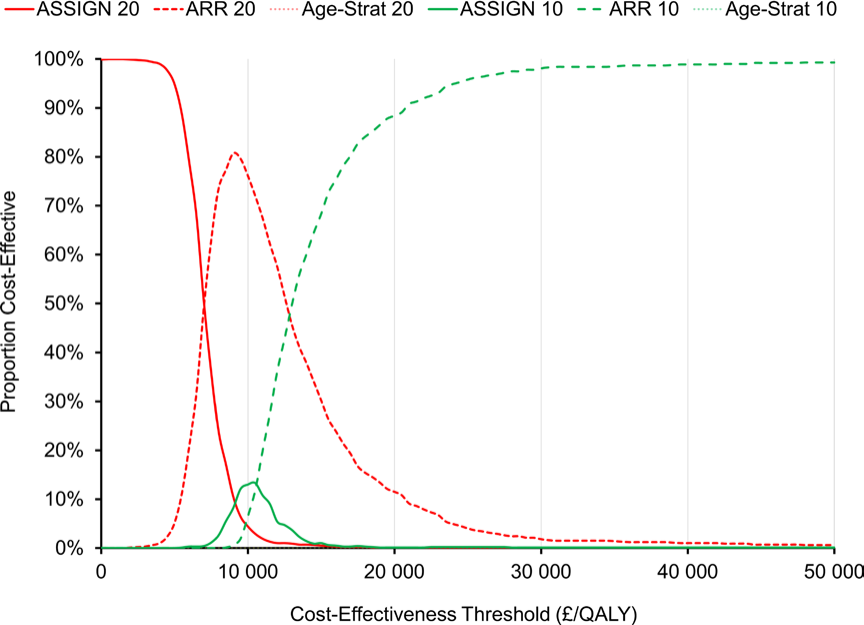
**Cost-effectiveness acceptability curves for all treatment strategies.** The curves for age-stratified risk threshold 10 and age-stratified risk threshold 20 are indistinguishable from the 0% line. ARR indicates absolute risk reduction; ASSIGN 10, individuals with an ASSIGN score ≥10%; ASSIGN 20, individuals with an ASSIGN score ≥20; and QALY, quality-adjusted life-year.

At cost-effectiveness thresholds <£50 000/QALY, ARR-based prioritization was optimal in most probabilistic iterations of the model (Figure 3). When all treatment strategies were considered together, ARR 10 was optimal 88% of the time at a threshold of £20 000/QALY.

**Figure 4.**
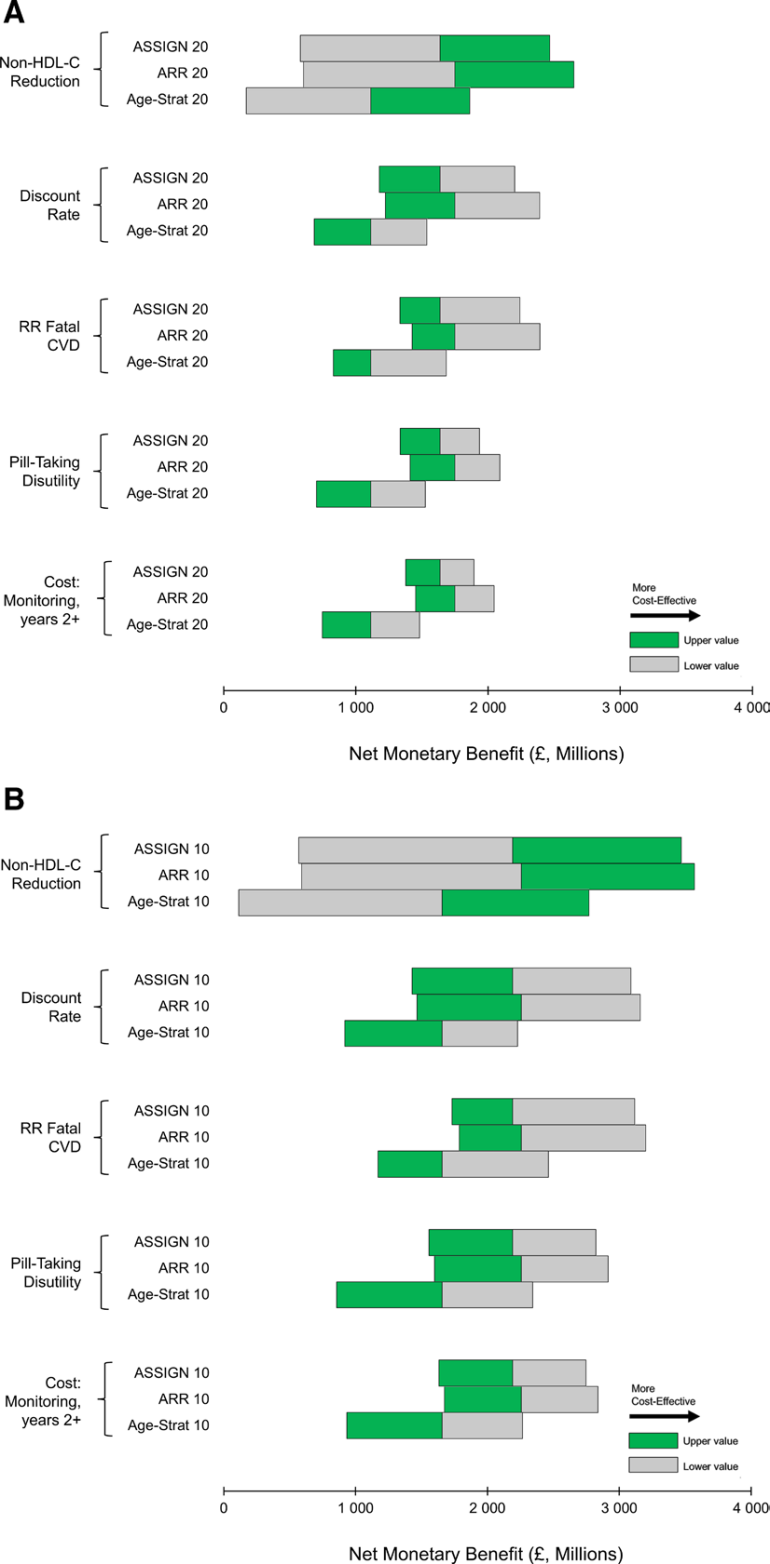
**Tornado diagrams for the most influential model measures. A**, Strategies treating the same number as individuals with an ASSIGN score ≥20 (ASSIGN 20). **B**, Strategies treating the same number as individuals with an ASSIGN score ≥10% (ASSIGN 10). Quality-adjusted life-years valued at £20 000. Increased net monetary benefit indicates increased cost-effectiveness. ARR indicates absolute risk reduction; CVD, cardiovascular disease; HDL-C, high-density lipoprotein cholesterol; and RR, relative risk.

Reduction in non–HDL-C, discount rate, the effect of statins on CVD mortality, pill-taking disutility, and ongoing monitoring costs had the greatest effect on cost-effectiveness estimates (Figure 4 and Figures S6 and S7). ARR remained the optimal approach to statin prioritization in most sensitivity analyses. However, at high levels of pill-taking disutility, treating fewer individuals was optimal (ie, ARR 20 had greater net monetary benefit than ARR 10; Figure S8). Neither accounting for reduced patient adherence nor removing patients with diabetes from the simulation cohort greatly affected cost-effectiveness results, with ARR 10 remaining the optimal treatment strategy at a cost-effectiveness threshold of £20 000/QALY in both these analyses (Tables S13 and S14).

## DISCUSSION

The objectives of this study were to estimate the cost-effectiveness of expanding statin eligibility in Scotland and to estimate the cost-effectiveness of novel approaches to statin prioritization, relevant to all nations. Expanding statin eligibility in Scotland would be cost-effective and population health would be improved by using ARR, on the basis of both 10-year CVD risk and non–HDL-C level, to guide statin treatment decisions. Therefore, guideline committees should consider new ways to allocate statins on the basis of ARR and not simply 10-year risk thresholds.

ARR may be more clinically acceptable than 10-year risk scoring. Some physicians were dismayed by the decision of the National Institute for Health and Care Excellence to reduce the risk threshold for statin initiation to 10%.^44^ Polypharmacy, labeling, and treatment of relatively healthy individuals and potential adverse events were cited as concerns. These concerns may partly explain poor clinical adherence to statin guidelines.^45^ The ARR approach treats patients with measurably unhealthy levels of a modifiable risk factor and may be more palatable to clinicians.

The benefits associated with age-stratified risk thresholds may also have been underestimated. Atherosclerosis is a cumulative process. Reducing exposure to risk factors that exacerbate atherosclerotic buildup in early life will have an outsized effect on averting later life CVD events.^46,47^ We did not account for cumulative exposure to risk factors in our analysis and estimated statin-related risk reduction from medium-term cardiovascular outcomes trials. Accounting for cumulative exposure would require access to a longitudinal data set that regularly tracked participants’ risk factors. Such a data set does not exist for the general Scottish population. Combining information on patients’ lifetime CVD risk and current cholesterol levels may allow clinicians to better target treatment at younger patients with a high capacity to gain from early statin initiation.^48,49^

In a validation exercise, the Scottish CVD Policy Model simulated individuals from the placebo and treatment arms of the West of Scotland Coronary Prevention Study (Figures S1 and S2). The model was well-matched to CVD event rates in most comparisons. However, rates of CHD were lower in the simulated placebo arm compared with trial data. If the model systematically underestimates CVD event rates in the untreated CVD-free population, intensive lipid-lowering strategies will be more cost-effective than simulated. Event rates in the model were recalibrated to replicate contemporary Scottish life tables, which should ensure a better fit with contemporary CVD event rates than observed in the validation exercise.^19^

As with all decision modeling studies, uncertainty in model measures propagates into uncertainty in modeled outcomes. Whereas the efficacy and side effects of statins have been studied extensively, more research could be conducted to assess statin pill-taking disutility and establish optimal treatment monitoring procedures. Pill-taking disutility is a loosely defined and underresearched phenomenon, with most available evidence coming from small sample studies and online surveys of select populations.^50,51^ The results of our sensitivity analyses suggest that benefits of statins do not outweigh the costs for patients who are highly averse to taking pills regularly. The necessary frequency of patient monitoring also likely varies substantially between patients. Our results show that reducing monitoring costs greatly increases the cost-effectiveness of statin therapy.

### Conclusions

The advent of generic pricing has rendered preventive statin therapy cost-effective for many adults in Scotland. Eligibility for statin therapy should be expanded to ensure that more individuals who could benefit from statins are treated. Novel mechanisms for statin prioritization may further improve population health. ARR synthesizes information on a patient’s absolute CVD risk with RRR from statin therapy; it is a cost-effective approach to statin prioritization and may be more appealing to clinicians than recommendations solely on the basis of 10-year risk thresholds.

## Article Information

### Sources of Funding

Dr Kohli-Lynch is supported by the Medical Research Council, Swindon (grant MR/K501335/1) and the National Institute for Disability, Independent Living, and Rehabilitation Research (grant 610-5441030-60057402). Dr Sattar is supported by the British Heart Foundation Research Excellence Award (RE/18/6/34217). The Article Processing Charge was paid by the University of Glasgow open access research fund. The sponsors had no role in the design or conduct of the study; collection, management, analysis, or interpretation of the data; preparation, review, or approval of the manuscript; or decision to submit the manuscript for publication.

### Disclosures

Dr Sattar discloses personal fees from Afimmune, Amgen, AstraZeneca, Boehringer Ingelheim, Eli Lilly, Hanmi Pharmaceuticals, Merck Sharp and Dohme, Novartis, Novo Nordisk, Pfizer, and Sanofi; and grant funding paid to his university from AstraZeneca, Boehringer Ingelheim, and Roche Diagnostics. The other authors report no conflicts.

### Supplemental Material

Formulating Absolute Risk Reduction

Scottish Cardiovascular Disease Policy Model

Defining Alternative Prioritization Criteria

Simulation Cohort

Figures S1–S8

Tables S1–S14

References 52–72

## Supplementary Material


