# Supplementary material for: Beyond 10-Year Risk: A Cost-Effectiveness Analysis of Statins for the Primary Prevention of Cardiovascular Disease
Source: Circulation. 2022 Mar 7;145(17):1312–23. doi: 10.1161/CIRCULATIONAHA.121.057631 (PMC9022692; doi:10.1161/CIRCULATIONAHA.121.057631)

SUPPLEMENTAL MATERIAL

Title: Beyond Ten-Year Risk: A Cost-Effectiveness Analysis of Statins for the Primary Prevention of Cardiovascular Disease

Table of Contents

1. **Investigator List**
2. **Supplement.** Formulating Absolute Risk Reduction. Scottish CVD Policy Model. Simulation Cohort.
3. **eTable 1:** Cause-specific hazards of primary events in the Scottish CVD Policy Model
4. **eTable 2:** Cause-specific hazards of post-CVD mortality in the Scottish CVD Policy Model
5. **eTable 3:** Probit regression, probability of secondary non-fatal CVD events
6. **eTable 4:** Linear regression, utility decrements from various CVD events
7. **eTable 5:** Linear regression, pre-event costs
8. **eTable 6:** Linear regression, post-event costs
9. **eTable 7:** Descriptive statistics, SHS 2011 participants who refused nurse visits
10. **eTable 8:** Risk assessment, monitoring, and side effect costs of statin therapy
11. **eTable 9.** Consolidated Health Economic Evaluation Reporting Standards statement
12. **eTable 10:** Descriptive statistics of patient populations
13. **eTable 11:** Cost-effectiveness results, multiple ASSIGN score thresholds
14. **eTable 12:** Disaggregated cost of different policies
15. **eTable 13:** Cost-effectiveness results – reduced statin therapy adherence
16. **eTable 14**: Cost-effectiveness results – omitting individuals with diabetes from cohort
17. **eFigure 1:** Predicted versus observed primary events in placebo arm of the WOSCOPS trial
18. **eFigure 2:** Predicted versus primary events in treatment arm of the WOSCOPS trial
19. **eFigure 3:** Proportion of population eligible for treatment with different risk thresholds
20. **eFigure 4:** Proportion of age-groups eligible for treatment with different risk thresholds
21. **eFigure 5:** Proportion of population eligible for treatment with different ARR thresholds
22. **eFigure 6:** Tornado diagrams showing sensitivity analysis results
23. **eFigure 7:** Graphical representation of one-way sensitivity analysis results
24. **eFigure 8:** Net monetary benefit of treatment strategies versus pill-taking disutility

Title: Beyond Ten-Year Risk: A Cost-Effectiveness Analysis of Statins for the Primary Prevention of Cardiovascular Disease

Authors:
Ciaran N. Kohli-Lynch, PhD[1,2]; James Lewsey, PhD[2]; Kathleen A. Boyd, PhD[2]; Dustin D. French, PhD[1,3,4]; Neil Jordan, PhD[1,3,5]; Andrew E. Moran, MD[6]; Naveed Sattar, PhD[7]; David Preiss, PhD[8]; Andrew H. Briggs, PhD[2,9]

Affiliations:
1. Center for Health Services and Outcomes Research, Northwestern University, Chicago, IL, USA
2. Health Economics and Health Technology Assessment, University of Glasgow, Glasgow, UK
3. Center of Innovation for Complex Chronic Healthcare, Hines VA Hospital, Hines, IL, USA
4. Department of Ophthalmology and Medical Social Science, Feinberg School of Medicine, Northwestern University, Chicago, IL, USA
5. Departments of Psychiatry & Behavioral Sciences and Preventive Medicine, Feinberg School of Medicine, Northwestern University, Chicago, IL, USA
6. Division of General Medicine, Columbia University Irving Medical Center, New York, USA
7. Institute of Cardiovascular and Medical Sciences, University of Glasgow
8. Medical Research Council Population Health Research Unit, Clinical Trial Service Unit and Epidemiological Studies Unit, Nuffield Department of Population Health, University of Oxford, Oxford, United Kingdom
9. Department of Health Services Research and Policy, London School of Hygiene and Tropical Medicine

Correspondence to:
Ciaran N. Kohli-Lynch, PhD
Center for Health Services & Outcomes Research
Northwestern University
633 N. St. Clair St.
Suite 2000
Chicago, IL 60611
ciaran.kohli-lynch@northwestern.edu

Reference list included in main paper

1. Formulating Absolute Risk Reduction

Thanassoulis et al. developed an equation to predict 10-year absolute risk reduction (ARR) attributable to statin therapy.[52] This equation accounts for both absolute 10-year risk (R) and baseline low-density lipoprotein cholesterol (LDL-C). The following section aims to formalize the definitions of risk and benefit employed in our analysis and describe the equations from Thanassoulis et al. that we used to estimate individual-level ARR from statin therapy.

Let R_{un} equal baseline (untreated) 10-year risk and S_{un} be baseline 10-year cardiovascular disease (CVD)-free survival. An individual's baseline cumulative hazard, H_{un}, is equal to their cumulative instantaneous hazard, *h*, from initial observation until time *t* (Equation 1-1). The relationships between risk, survival and hazard are presented in Equations 1-2 to 1-4.[53]

$$H_{un} = \int_0^t h\,dt \tag{1-1}$$

$$S_{un} = 1 - R_{un} \tag{1-2}$$

$$R_{un} = \exp\,(-H_{un}) \tag{1-3}$$

$$H_{un} = -\ln(1 - R_{un}) = -\ln\,(S_{un}) \tag{1-4}$$

If *HR* is the hazard ratio associated with a 1.0 mmol/L reduction in LDL-C and ldl_b is an individual's baseline LDL-C, then the hazard ratio associated with a 40% reduction in LDL-C caused by statin therapy can be described by Equation 1-5.

$$x = HR^{ldl_b * 40\%} \tag{1-5}$$

Assuming proportional hazards, statins continuously reduce an individual's instantaneous hazard of experiencing a CVD event. Applying the hazard ratio *x* from Equation 1-5 to an individual's instantaneous hazard function enables estimation of treated cumulative hazard, H_{tr}, as shown in Equation 1-6. Evidently, there is a direct relationship between treated and untreated cumulative hazard. Combining Equation 1-4 and Equation 1-6, we can establish the relationship between event-free survival with and without treatment.

$$H_{tr} = \int (x * h)\,dt = x \int_0^t h\,dt = x * H_{un} \tag{1-6}$$

$$S_{tr} = {S_{un}}^{x} \tag{1-7}$$

Absolute risk reduction from statin therapy is the difference in absolute risk with and without treatment, as presented in Equation 1-8. Combining Equation 1-2, Equation 1-7, and Equation 1-8 ultimately produces the equation used to estimate ARR in our analysis (Equation 1-9). The value of HR can be derived from meta-analyses of statin trials. Hence, an individual's ARR can be calculated as a function of their R_{un} (estimated using a 10-year CVD risk score) and ldl_b. Estimates of HR derived from clinical trials are potentially biased as trial exclusion criteria can produce clinical trial cohorts that are not representative of the target population for a treatment.[54]

$$\text{ARR} = R_{un} - R_{tr} \tag{1-8}$$

$$\text{ARR} = {S_{un}}^{x} - S_{un} \tag{1-9}$$

2. Scottish CVD Policy Model

Background

In 2010 the Chief Scientist Office for Scotland funded research to develop the Scottish CVD Policy Model.[24] This is a decision-analytic model that predicts life expectancy (LE), quality-adjusted life expectancy (QALE), and cost outcomes for individuals based on their ASSIGN risk factors.[19,20] It was originally developed as two extensive Microsoft Excel files, one for males and one for females. In 2021, the model was redeveloped in the R computing language and an open-source copy is now available online at https://github.com/yiqiaoxin/CVDmodel.[55]

Structure

A diagram of the model is presented in **Manuscript Figure 1**. Individuals enter CVD-free, and transition to one of four first event types throughout the course of their lives: non-fatal coronary heart disease (CHD), non-fatal cerebrovascular disease (CBVD), fatal CVD, or fatal non-CVD.

Each state in the model has an assigned quality-adjusted life year (QALY) value. Individuals who have not experienced a primary CVD event are attributed a background QALY value. Individuals inhabiting one of the two non-fatal chronic CVD states are assigned a decrement to their background QALY value, determined by the type of first event (CHD or CBVD). Within the chronic disease states, a proportion of individuals experience further utility decrements attributable to secondary CVD events. Costs are also assigned to all health states in the model.

The sources and methodology used to derive health and cost estimates for each state in the model are described below.

Simulation

The Scottish CVD Policy Model can be employed in two ways. Primarily, it can produce individual-level outcome estimates for prospective patients based on their ASSIGN risk factors. In turn this can inform patient and physician decision-making.

Individual-level outcome estimates are obtained by inputting the individual's risk factor information into the model. These factors then dictate the probabilities that the individual will inhabit a given model state in each cycle of analysis. These factors also determine the cost and QALYs that an individual will accumulate in each state and cycle combination. The individual's expected cost and QALYs are then summed over the time horizon of the analysis, as shown in Equation 2-1 and Equation 2-2, respectively.

$$E[c] = \sum_{t=0}^{h} \sum_{s} p_{s,t} * c_{s,t} \quad (2\text{-}1)$$

$$E[e] = \sum_{t=0}^{h} \sum_{s} p_{s,t} * e_{s,t} \quad (2\text{-}2)$$

E[c] and *E[e]* are the expected cost and expected health effects, respectively. These values are equal to the product of an individual's probability of inhabiting disease state, *s*, in cycle, *t*, summed over the time horizon of the analysis, *h*, and all disease states included in the model.

A further use of the model is to estimate population-level outcomes. In this situation, individual-level outcome estimation is computed as described above for a cohort of individuals. The risk factor profiles for this analysis can be derived from large-scale cross-sectional surveys like the Scottish Health Survey.[26] These outcomes can then be projected onto a wider population. This was the approach adopted in our analysis of statin cost-effectiveness.

<u>State Transition Probabilities</u>

Two types of state transitions exist in the model: transition to a primary event (fatal or non-fatal) and transition to the all-cause mortality absorbing state after a primary non-fatal event. Both these transitions are determined by equations derived from competing risk survival analysis of a longitudinal dataset of Scottish adults.

All state transition probabilities in the model are derived from a dataset that linked baseline risk factor information in the Scottish Heart Health Extended Cohort[22,22,56] to a collection of routinely collected clinical data called the Scottish Morbidity Records (SMR).[23]

The SHHEC is an extensive dataset that was used in the construction of the ASSIGN score. Baseline CVD risk factor information was recorded for 6,419 men and 6,618 women from 25 Scottish districts between 1986 and 1995. The risk factors collected at baseline were age, sex, total cholesterol (TC), high-density lipoprotein cholesterol (HDL-C), systolic blood pressure (SBP), family history of CVD, diabetes, cigarettes smoked per day (CPD), and Scottish Index of Multiple Deprivation (SIMD). These will be referred hereafter as the *ASSIGN risk factors*.

The SMR is a database maintained by the Information Services Division (ISD) of the Scottish National Health Service (NHS). This database records all hospitalized events that occur in the Scottish NHS, detailing reason for admission, up to five secondary diagnoses, length of stay, and wait time. Health boards submit hospitalisation data to the ISD every six weeks, and audits of the SMR have found its data to be 99% complete.[23,57]

SHHEC participants agreed to have their baseline data linked with the SMR via their unique NHS patient identification numbers. Linking these two datasets allows researchers to analyse the relationship between individuals' baseline characteristics and health outcomes. The most recent SHHEC-SMR linkages included data through 2006 and 2009, respectively. The 2006 linkage was employed in the development of the ASSIGN score and the 2009 linkage was employed in the development of the Scottish CVD Policy Model.

A parametric competing risks approach was adopted to estimate the probability of primary events.[58] The competing risk approach provides an unbiased methodology for estimating event probability in the presence of competing events. Primarily, it involved estimating cause-specific hazard functions for a set of mutually exclusive events. Probability of event-free survival can then be computed as a function of survival from all event types.

Cause-specific hazard reflects the probability that an individual will experience an event at a moment in time, conditional on the fact that they have not yet experienced a competing event. It can be estimated with parametric regression analysis. This analysis models the functional form of a population's cause-specific hazard based on some pre-defined statistical distribution. Gompertz regression[59] was performed on the SHHEC-SMR dataset using Stata software (Version 12.1 StataCorp LP)[60] to estimate cause-specific hazard functions for the four primary events in the model: non-fatal CHD, non-fatal CBVD, fatal CVD, and fatal non-CVD. All ASSIGN variables were included as covariates in these regression models. Primary event regression equations, developed by Lewsey et al., are presented in **eTable 1**.[19]

Transition rates from the two non-fatal CVD states to all-cause mortality were derived by conducting Gompertz regression on the SHHEC-SMR dataset. Competing risks were not relevant as the analysis only considered the probability of transition to an absorbing state with no competing events. The covariates employed in this regression were age at first event, SIMD, and family history of CVD. Results for the secondary event regressions, also developed by Lewsey et al., are presented in **eTable 2**.[19]

Each year individuals in the chronic disease states were at risk of experiencing angina, myocardial infarction, irregular heartbeat, other heart condition, stroke, and intermittent claudication. The proportion of individuals within chronic disease states experiencing these events was computed with probit regression (**eTable 3**). The covariates used in these regressions were age at first event, SIMD, and family history of CVD. Cubic splines were included in the regression model to reflect non-linearity over time. Probability-weighted utility decrements and costs were assigned to chronic disease states to capture the effect of these secondary events.

Health-Related Quality of Life

Health-related quality of life (HRQoL) was assigned to disease states based on analysis of the Scottish Health Survey (SHS) 2003.[61] This is the most recent largescale survey of the Scottish population to measure quality of life. A total of 7,054 survey respondents aged ≥20 years completed 12-item Short Form (SF-12) HRQoL questionnaires, which can be used to generate QALYs.[62]

Linear regression was conducted on SHS 2003 data to produce baseline QALY estimates for the adult Scottish population and to estimate utility decrements associated with a range of CVD events. The dependent variable in this regression was SF-12-derived QALY value and the

independent variables were sex, age, and six CVD events. The results from this analysis are shown in **eTable 4**.

Healthcare Costs

Lifetime hospitalisation costs were estimated for all individuals in the SHHEC-SMR dataset. These were estimated as a function of the events experienced by an individual and overall length of stay. The costed SHHEC-SMR dataset was then analyzed to predict health state costs in the model.

Method 1 from Geue et al.[63] was employed to attribute costs of continuous inpatient stays (CIS) to each hospitalisation episode observed in the SHHEC-SMR dataset. This required assigning a healthcare resource group (HRG) to each hospitalisation episode in the dataset with HRGv3.5 Grouper software[64], followed by attributing costs to these episodes from the English NHS tariff.[65] Finally, it was necessary to estimate the overall cost of each CIS that involved more than one episode of care. Treating all episodes of care separately would lead to overestimation of costs, so an approach was adopted which established a dominant episode (and HRG) for all hospitalisations, but which simultaneously accounted for other non-dominant episodes of care within this CIS. This was achieved by using a ‘Spell Converter’ software which designates episode of care dominance based on admission date, discharge date, episode order, length of stay, and HRG.

Once lifetime hospitalisation costs were estimated for every individual in the SHHEC-SMR dataset, these data were employed in linear regression models to predict pre- and post-event hospitalisation costs in the model (**eTables 5-6**). Cubic splines were included in the regression model to reflect non-linearity over time in the cost estimations. Other covariates included were age at model entry (for pre-event costs), age at first event (for post-event costs), SIMD, and family history of CVD.

Discrimination and Calibration

Internal and external validation of the model has been completed. These validation exercises have shown that the model has a good level of discrimination and calibration.[24]

Internal validation of the functions that dictate state transition in the model was completed by means of AUROC analysis. The c-statistics for the primary risk functions for men and women are detailed in eTable 1. For primary events they range from 0.70-0.80, and for mortality post non-fatal CHD and CBVD they range from 0.65-0.68. C-statistics between 0.70 and 0.80 are considered to provide “acceptable” discrimination for models of CVD, according to Lloyd-Jones, and a score of 0.65 performs “much better than random chance”.[66]

The calibration of the model was assessed with data from the West Of Scotland COronary Prevention Study (WOSCOPS).[67] WOSCOPS was a placebo-controlled trial of pravastatin which

enrolled men aged between 45 and 64 years with hypercholesterolaemia, with an initial 5-year follow-up.

The baseline data of men in the placebo and treatment arms of WOSCOPS were inserted into the Scottish CVD Policy Model. Predicted cumulative incidence of non-fatal CHD, non-fatal CBVD, fatal CVD, and fatal non-CVD was recorded. These results were then visually compared to event rates in the WOSCOPS population. The results of this analysis compared to the placebo arm of the trial are presented in **eFigure 4**. The results for the treatment arm are shown in **eFigure 5**.

Empirical data and simulation output were relatively close for the placebo and treatment arms of the trial. For patients who received placebo, non-fatal CBVD, fatal CVD, and fatal non-CVD events, predicted cumulative incidence generally fell within the confidence interval of observed results. However, the model systematically underpredicted incidence of non-fatal CHD. Results were more promising for the treatment arm of the trial. This suggests that the model can reliably assess the impact of primary interventions on CVD incidence in the Scottish population. The lack of complete agreement between the model and external data, however, serves as a reminder that the model is not able to perfectly predict outcomes in the Scottish population and that it is necessary to explore uncertainty in any results that the model produces.

<u>Recalibration</u>

The Scottish CVD Policy Model was built with data that is becoming increasingly outdated. Event rates for CVD have followed a continuous downwards trajectory in Scotland since the middle of the 20th century.[68] This reduction in event rate has been attributed to changes in biologic, demographic, and sociodemographic risk factors alongside improvements in health technology. Risk functions developed with data from the past likely overestimate CVD incidence. We recalibrated the risk functions in the model to better reflect current health outcomes in Scotland.

Risk functions in the model were recalibrated to replicate contemporary Scottish life tables. This Recalibration was achieved by multiplying the linear predictor in the risk functions by a set of multiplicative factors and recording predicted LE for a range of risk profiles. Predicted LE was then compared with 2020 Scottish life tables.[69] The multiplicative factor which produced the smallest root mean square error (RMSE) between predicted LE and life tables was employed in the model. This process was completed for the male and female models separately. Ultimately, recalibration led the RMSE to be reduced from 1.54 to 0.57 years for men and from 2.05 to 0.60 years for women.[19,24]

3. Defining Alternative Prioritization Criteria

Age-Stratified Risk Thresholds

Age-stratified risk thresholds were defined by splitting individuals into five-year age-groups from ages 40 to 79 and a group for individuals aged ≥80 years. For Age-Strat 20, we observed the proportion of the CVD-free adult population eligible for statins under ASSIGN 20 in SHS 2011 and set separate risk thresholds for each age-group which would treat this proportion of patients. Hence, age-stratified risk thresholds targeted treatment at individuals who were high-risk relative to their age-group peers. This process was also used to define an age-stratified risk policy which treats the same proportion of the population as ASSIGN 10.

Absolute Risk Reduction Thresholds

We estimated ARR from statins for everyone in the SHS cohort using a modified form of **Equation 1**. Due to data limitations, LDL-C was replaced with non-HDL-C. The percentage reduction in non-HDL-C and relative risk per 1.0 mmol/L reduction were altered in the equation accordingly. Statins were assumed to reduce non-HDL-C by 26.0%.[17] The relative risk of CVD associated with a 1.0 mmol/L reduction in non-HDL-C was estimated to be 0.79. We established ARR thresholds that would treat the same proportion of the population as ASSIGN 20 and ASSIGN 10, respectively.

4. Simulation Cohort

The simulation cohort was developed with a combination of Scottish Health Survey (SHS) 2011 data[26] and contemporary population estimates from the National Records of Scotland.[25] Both these data sources are publicly available through the U.K. Data Service and National Records of Scotland websites, respectively.

The SHS is a study of public health which was commissioned by the Scottish Government Health Directorates.[26] It was conducted face-to-face with trained interviewers, contains information on many health indicators, and is principally focused on CVD. Values for all ASSIGN risk factors can be derived for all survey respondents from SHS data.

The survey used a multi-stage stratified probability sampling design.[70] Data were obtained from 25 strata: the three island health boards (Orkney, Shetland, and Western Isles), along with 22 other groups constructed by dividing the remaining 11 Scottish health boards into data zones containing "deprived" and "non-deprived" populations. Areas were deemed to be deprived if they were in the top 15% of deprived areas according to SIMD. Stratification allowed for the oversampling of deprived areas. This was to ensure the survey gave a representative sample of the Scottish population, as response rates for surveys are typically lower in deprived areas.

In 2011, the SHS consisted of two stages. All respondents completed an initial interview which obtained information on core topics including household information, general health, general CVD, use of health services, lifestyle factors, economic activity, education, ethnic background, national identity and origin, family health background, and height and weight. The second stage was a nurse interview, in which blood samples were obtained. A subsample of those interviewed in Stage 1 was offered nurse interviews. During nurse visits, information on patients' cholesterol levels and blood pressure were recorded.

Additional data were needed to project results onto the Scottish population. Mid-year population estimates from the National Records of Scotland[25,26] provided information on the size of the Scottish population and the distribution of age-groups within it.

Multiple Imputation

The SHS 2011 provided information on all risk factors required for simulation with the Scottish CVD Policy Model. In total, 10,431 addresses were selected for initial sample of the SHS 2011. Interviews were conducted with 7,544 adults and the estimated response rate was approximately 56%. In total 4,644 respondents were aged ≥40 years with no established CVD and were included in our final dataset.

The SHS dataset can be split into four subsets: individuals with complete ASSIGN risk factor information (n=413), individuals who completed a nurse interview but had some remaining missing covariate information (n=201), individuals who were not offered a nurse interview so

provided no TC, HDL-C, or SBP information (n=3,724), and individuals who refused a nurse interview (n=306). Of the 201 individuals who completed the nurse interview but had some missing information, 141 (70.1%) did not have a recorded SBP reading and 60 (29.9%) did not have recorded cholesterol information.

We did not exclude individuals currently receiving statins (n=96) from our dataset. Instead, these individuals were 'detreated', by modifying cholesterol levels according to treatment effects observed in randomised trials of statin therapy.[17] Meta-analysis evidence shows statins reduce TC by approximately 19.8% and increase HDL-C by approximately 2.98%. Taking the inverse of these numbers, we assumed that detreating individuals would lead to a 24.7% increase in observed TC and a 2.89% reduction in HDL-C.

A key issue with the SHS data is the relatively small number of respondents for whom nurse interviews were performed. This means that data are sparse for three important modifiable CVD risk factors: TC, HDL-C, and SBP. We opted not to conduct a complete case analysis due to the small number of individuals who provided full risk factor profiles due to the potential for small sample bias. This problem would be exacerbated by the fact our analysis was stratified by age-group. In the older age-groups, CVD was widespread. For example, CVD prevalence in the dataset for individuals aged ≥80 years was 48%. In this age-group, blood sample information was available for only 25 individuals with no established CVD.

Complete information was available for respondents' age, sex, SIMD, diabetes status, and family history of CVD. Data on hours exercised per week was also available for all survey participants. Evidence suggests that exercise is strongly predictive of TC, HDL-C, and SBP.[71] For individuals who did not refuse a nurse visit, missing SBP, TC, and HDL-C values were imputed using all available ASSIGN variables plus weekly hours of exercise as predictors. These covariates were imputed with stochastic regression[27] to ensure the variation observed in the larger population was propagated into the imputed dataset.

For individuals who refused nurse visits, SBP, TC, and HDL-C values were estimated through multiple imputation.[28] Janssen et al.[72] show that imputing missing data is a more reliable means of obtaining unbiased estimates than removing variables with missing data or performing a complete case analysis in medical research. This result was validated even with 90% missing data in some variables, but strictly relied on the assumption that data were missing at random.

For the 306 individuals who refused a nurse visit, SBP, TC, and HDL-C, ten imputed risk profiles were created by with Stata software (Version 12.1, StataCorp LP).[60] Non-missing ASSIGN variables were employed in the imputation process along with the individual's weekly hours of exercise. During the simulation process, each of the ten imputed risk factor profiles was inputted into the model to simulate statin therapy, and the outcomes from these simulations were averaged to determine a central estimate of treatment effect health and cost outcomes. Descriptive statistics for individuals who refused a nurse visit are included in **eTable 7**.

Supplemental Tables

eTable 1: Cause-specific hazards of primary events in the Scottish CVD Policy Model

Covariate	Hazard ratio, non-fatal CHD	Hazard ratio, non-fatal CBVD	Hazard ratio, CVD mortality	Hazard ratio, non-CVD mortality	Source
**Men**					
Age (years)	1.05 (1.04, 1.05)	1.07 (1.06, 1.08)	1.10 (1.09, 1.11)	1.10 (1.09, 1.11)	SHHEC-SMR Dataset
SIMD	1.04 (1.01, 1.07)	1.10 (1.05, 1.15)	1.07 (1.03, 1.10)	1.10 (1.07, 1.13)	
Diabetes	1.93 (1.07, 2.76)	3.22 (1.94, 5.33)	2.37 (1.48, 3.81)	1.40 (0.84, 2.31)	
FH	1.50 (1.34, 1.69)	0.98 (0.79, 1.21)	1.18 (1.00, 1.39)	0.99 (0.85, 1.14)	
CPD	1.42 (1.34, 1.55)	1.61 (1.40, 1.86)	1.87 (1.67, 2.10)	1.84 (1.68, 2.02)	
SBP (mm Hg)	1.08 (1.31, 1.11)	1.12 (1.08, 1.17)	1.16 (1.13, 1.20)	0.99 (0.95, 1.02)	
TC (mmol/L)	1.29 (1.05, 1.35)	1.09 (1.00, 1.18)	1.13 (1.05, 1.21)	0.95 (0.90, 1.01)	
HDL-C (mmol/L)	0.68 (1.23, 0.75)	0.94 (0.82, 1.07)	0.93 (0.83, 1.04)	1.21 (1.11, 1.32)	
c-statistic	0.70 (0.62, 0.71)	0.73 (0.71, 0.75)	0.77 (0.76, 0.79)	0.74 (0.72, 0.75)	
**Women**					
Age (years)	1.06 (1.05, 1.07)	1.08 (1.07, 1.10)	1.11 (1.09, 1.12)	1.09 (1.08, 1.11)	SHHEC-SMR Dataset
SIMD	1.09 (1.06, 1.12)	1.14 (1.09, 1.19)	1.04 (1.00, 1.09)	1.08 (1.04, 1.11)	
Diabetes	2.07 (1.41, 3.03)	3.01 (1.81, 4.99)	3.14 (1.97, 5.00)	0.96 (0.51, 1.81)	
FH	1.68 (1.48, 1.90)	1.43 (1.16, 1.75)	1.27 (1.05, 1.53)	0.98 (0.85, 1.14)	
CPD	1.51 (1.34, 1.71)	1.71 (1.41, 2.08)	2.61 (2.24, 3.03)	2.14 (1.91, 2.41)	
SBP (mm Hg)	1.06 (1.03, 1.10)	1.15 (1.09, 1.20)	1.19 (1.14, 1.24)	1.03 (0.99, 1.06)	
TC (mmol/L)	1.21 (1.15, 1.27)	0.95 (0.86, 1.05)	1.06 (0.98, 1.15)	0.93 (0.87, 0.99)	
HDL-C (mmol/L)	0.69 (0.63, 0.76)	0.84 (0.73, 0.97)	0.92 (0.81, 1.04)	0.98 (0.89, 1.07)	
c-statistic	0.74 (0.73, 0.75)	0.76 (0.73, 0.78)	0.80 (0.78, 0.82)	0.72 (0.70, 0.74)	

CBVD – cerebrovascular disease, CHD – coronary heart disease, CPD – cigarettes per day, CVD – cardiovascular disease, HDL-C – high-density lipoprotein cholesterol (HDL-C), SHHEC – Scottish Heart Health Extended Cohort, SIMD – Scottish Index of Multiple Deprivation, SMR – Scottish Morbidity Records

eTable 2: Cause-specific hazards of post-CVD mortality in the Scottish CVD Policy Model

Covariate	Hazard ratio, mortality post-CHD	Hazard ratio, mortality post-CBVD	Source
**Men**			
Age at first event (years)	1.08 (1.07, 1.09)	1.07 (1.05, 1.09)	SHHEC-SMR Dataset
SIMD	1.14 (1.09, 1.19)	1.09 (1.03, 1.16)	
FH	0.97 (0.79, 1.18)	1.06 (0.77, 1.47)	
c-statistic	0.68 (0.65, 0.71)	0.65 (0.61, 0.69)	
**Women**			
Age at first event (years)	1.08 (1.06, 1.09)	1.07 (1.05, 1.09)	SHHEC-SMR Dataset
SIMD	1.08 (1.03, 1.13)	1.00 (0.93, 1.08)	
FH	0.75 (0.60, 0.95)	1.20 (0.86, 1.67)	
c-statistic	0.67 (0.63, 0.70)	0.66 (0.61, 0.71)	

CBVD – cerebrovascular disease, CHD – coronary heart disease, SHHEC – Scottish Heart Health Extended Cohort, SIMD – Scottish Index of Multiple Deprivation, SMR – Scottish Morbidity Records

eTable 3: Probit regression, probability of various secondary non-fatal CVD events within chronic disease states

Covariate	Probit regression coefficient					Source
	CHD	Stroke	Intermittent Claudication	Other Heart Condition	Heart Failure	
**Post-non-fatal CHD**						
**Men**						
t1	-0.019 (-0.040-0.002)	-0.101 (-0.147--0.055)	-0.049 (-0.104-0.006)	-0.06 (-0.091--0.028)	-0.152 (-0.195--0.11)	SHHEC-SMR Dataset
t2	0.071 (0.045-0.098)	0.098 (0.035-0.161)	0.034 (-0.039-0.107)	0.05 (0.008-0.092)	0.16 (0.098-0.223)	
Age at first event (years)	0.010 (0.005-0.016)	0.001 (-0.007-0.009)	-0.001 (-0.012-0.010)	0.005 (-0.002-0.013)	0.014 (0.004-0.023)	
SIMD	0.003 (0.001-0.005)	0.003 (0.000-0.007)	0.002 (-0.003-0.006)	0.003 (0.001-0.006)	0.002 (-0.002-0.006)	
Family history	0.106 (0.019-0.193)	-0.011 (-0.168-0.145)	-0.136 (-0.350-0.077)	0.174 (0.051-0.297)	0.044 (-0.121-0.21)	
Constant	-2.012 (-2.420--1.605)	-2.085 (-2.640--1.530)	-2.137 (-2.903--1.371)	-2.187 (-2.68--1.693)	-2.626 (-3.26--1.992)	
**Women**						
t1	-0.003 (-0.033-0.027)	-0.072 (-0.144-0.000)	-0.078 (-0.152--0.004)	-0.045 (-0.087--0.003)	-0.119 (-0.176--0.061)	SHHEC-SMR Dataset
t2	0.057 (0.011-0.103)	0.026 (-0.102-0.155)	0.106 (-0.015-0.228)	0.026 (-0.045-0.096)	0.133 (0.039-0.227)	
Age at first event (years)	0.010 (0.004-0.016)	0.007 (-0.006-0.019)	0.016 (-0.001-0.033)	0.012 (0.003-0.021)	0.018 (0.000-0.029)	
SIMD	0.001 (-0.001-0.004)	0.005 (0.001-0.009)	0.001 (-0.005-0.008)	0.001 (-0.002-0.004)	0.004 (-0.000-0.009)	
Family history	0.056 (-0.046-0.158)	-0.014 (-0.221-0.194)	-0.129 (-0.379-0.122)	-0.138 (-0.27--0.005)	-0.038 (-0.246-0.17)	
Constant	-2.137 (-2.58--1.694)	-2.638 (-3.492--1.783)	-3.274 (-4.558--1.990)	-2.407 (-3.00--1.81)	-3.017 (-3.587--2.177)	
**Post-non-fatal CBVD**						
**Men**						
t1	-0.069 (-0.150-0.012)	-0.035 (-0.087-0.017)	0.020 (-0.085-0.125)	-0.070 (-0.134--0.006)	-0.129 (-0.275-0.017)	SHHEC-SMR Dataset
t2	0.063 (0.049--0.174)	0.046 (-0.030-0.123)	-0.108 (-0.305-0.09)	0.077 (-0.016-0.17)	0.159 (-0.021-0.340)	
Age at first event (years)	-0.003 (-0.016-0.010)	0.010 (-0.001-0.021)	0.001 (-0.016-0.019)	0.004 (-0.009-0.017)	0.039 (0.009-0.070)	
SIMD	-0.002 (-0.008-0.004)	0.003 (0.000-0.006)	0.007 (0.001-0.014)	0.002 (-0.002-0.007)	-0.010 (-0.021-0.001)	
Family history	0.144 (-0.085-0.373)	0.019 (-0.154-0.191)	0.024 (-0.34-0.389)	0.053 (0.169-0.275)	0.353 (-0.063-0.77)	
Constant	-1.506 (-2.42--1.605)	-2.109 (-2.891--1.327)	-2.744 (-4.034--1.454)	-1.923 (-2.789--1.058)	-4.697 (-6.983--2.41)	
**Women**						
t1	0.078 (-0.028-0.183)	-0.023 (-0.077-0.03)	0.008 (-0.137-0.152)	-0.053 (-0.138-0.032)	-0.186 (-0.319--0.054)	SHHEC-SMR Dataset
t2	-0.088 (-0.225-0.049)	0.056 (-0.02-0.132)	-0.069 (-0.275-0.137)	0.034 (-0.071-0.140)	0.227 (0.069-0.386)	
Age at first event (years)	-0.000 (-0.014-0.013)	0.022 (0.013-0.030)	-0.011 (-0.031-0.009)	0.006 (-0.005-0.017)	-0.002 (-0.017-0.014)	
SIMD	0.004 (-0.003-0.011)	0.001 (-0.002-0.004)	0.000 (-0.013-0.014)	-0.001 (-0.006-0.004)	0.001 (-0.007-0.010)	
Family history	0.068 (-0.222-0.359)	0.042 (-0.192-0.108)	-0.303 (-0.798-0.192)	0.281 (0.044-0.517)	0.036 (-0.36-0.432)	
Constant	-2.531 (-3.653--1.409)	-2.777 (-3.424--2.130)	-1.714 (-3.543-0.115)	-2.178 (-2.983--1.373)	-1.881 (-3.219--0.542)	

CBVD – cerebrovascular disease, CHD – coronary heart disease, SHHEC – Scottish Heart Health Extended Cohort, SIMD – Scottish Index of Multiple Deprivation, SMR – Scottish Morbidity Records, t1 – time spline 1, t2 - time spline 2

eTable 4: Linear regression, utility decrements from various CVD events

Covariate	Utility Decrement (SE)
Age (years)	0.0005 (0.0001)
Male	-0.0240 (0.0030)
Angina	0.0890 (0.0070)
Myocardial infarction	0.0400 (0.0100)
Irregular heartbeat	0.0500 (0.0070)
Other heart condition	0.0340 (0.0120)
Stroke	0.0940 (0.0110)
Intermittent claudication	0.0200 (0.0100)
Constant	0.1770 (0.0050)

SE – standard error

eTable 5: Linear regression, pre-event costs

Covariate	Post-Event Costs, Linear Regression Coefficient				Source
	Non-fatal CHD	Non-fatal CBVD	Fatal CVD	Fatal non-CVD	
**Men**					
t1	19 (-2-39)	6 (-38-49)	26 (-7-59)	42 (-5-89)	SHHEC-SMR Dataset
t2	115 (70-160)	157 (72-241)	115 (57-173)	237 (157-317)	
Age at first event (years)	23 (16-29)	17 (6-29)	28 (17-29)	25 (9-41)	
SIMD	5 (3-7)	7 (3-10)	4 (0-8)	5 (-1-10)	
Family history	94 (2-186)	-162 (-329-5)	67 (121-256)	117 (-199-433)	
Constant	-1121 (-1446--795)	-833 (-1483--182)	-1345 (-1981--709)	-1029 (-1890--169)	
**Women**					
t1	-7 (-94-80)	14 (-23-52)	24 (-18-66)	59 (12-106)	SHHEC-SMR Dataset
t2	172 (-65-409)	122 (58-186)	145 (72-217)	203 (126-279)	
Age at first event (years)	1 (-25-26)	26 (15-38)	34 (18-49)	16 (-4-35)	
SIMD	11 (2-19)	8 (2-14)	6 (1-10)	12 (6-18)	
Family history	338 (-236-911)	23 (-177-222)	106 (-125-336)	48 (-230-325)	
Constant	-214 (-1359-931)	-1462 (-2123--	-1727 (-2643--810)	-832 (-1894-230)	

CBVD – cerebrovascular disease, CHD – coronary heart disease, SHHEC – Scottish Heart Health Extended Cohort, SIMD – Scottish Index of Multiple Deprivation, SMR – Scottish Morbidity Records, t1 – time spline 1, t2 - time spline 2

eTable 6: Linear regression, post-event costs

Covariate	Post-Event Costs, Linear Regression Coefficient	Source
**Post non-fatal CHD**		
**Men**		
t1	-553 (-639--467)	SHHEC-SMR Dataset
t2	655 (555-755)	
Age at first event (years)	85 (67-102)	
SIMD	14 (8-21)	
Family history	240 (-80-560)	
Constant	-1024 (-2107-59)	
**Women**		
t1	-549 (-652--445)	SHHEC-SMR Dataset
t2	745 (600-890)	
Age at first event (years)	91 (69-113)	
SIMD	14 (6-21)	
Family history	-228 (-597-141)	
Constant	-1321 (-2900-257)	
**Post non-fatal CBVD**		
**Men**		
t1	-680 (-855--505)	SHHEC-SMR Dataset
t2	788 (556-1020)	
Age at first event (years)	113 (81-144)	
SIMD	7 (-5-18)	
Family history	-102 (-717-513)	
Constant	-1836 (-4010-338)	
**Women**		
t1	-542 (-744--340)	SHHEC-SMR Dataset
t2	596 (357-834)	
Age at first event (years)	97 (67-127)	
SIMD	8 (-5-20)	
Family history	-94 (-656-468)	
Constant	-1251 (-3593-1092)	

CBVD – cerebrovascular disease, CHD – coronary heart disease, SHHEC – Scottish Heart Health Extended Cohort, SIMD – Scottish Index of Multiple Deprivation, SMR – Scottish Morbidity Records, t1 – time spline 1, t2 - time spline 2

eTable 7: Descriptive statistics for individuals in Scottish Health Survey 2011 who refused nurse visits; SBP, TC, and HDL-C values are the average across ten multiply imputed datasets

Risk Factor	Observations	Mean	Standard Deviation
**Male**	306	0.39	0.49
**Age (years)**	306	60.4	12.92
**SIMD**	306	22.1	14.65
**Diabetes**	306	0.09	0.29
**Family History**	306	0.48	0.50
**CPD**	306	7.00	3.77
**SBP (mmHg)**	306	133	8.83
**TC (mmol/L)**	306	5.6	0.53
**HDL-C (mmol/L)**	306	1.5	0.23
**ASSIGN Score**	306	23.1	20.1

HDL-C – high-density lipoprotein cholesterol, SBP – systolic blood pressure, TC – total cholesterol

eTable 8: Risk assessment, monitoring, and side effect costs of statin therapy

Resource	Risk Assessment	Year 1	Year 2+	Unit Price (£)	Source
**Appointments**					
Blood sample (with healthcare assistant)	1	2	1	4.23	
Appointment with nurse, 10 mins	1	0	1	7.00	
Appointment with GP, 10 mins	0	2.2	1	39.00	
**Blood tests**					
Total cholesterol	0	2	1	1.00	5,34,36,38
HDL cholesterol	0	2	1	1.00	
Combined lipid profile	1	0	0	3.00	
Liver transaminase (ALT or AST)	1	2	1	1.00	
Creatine kinase	0.1	0	0	2.00	
HbA1c	1	1	1	2.25	
**Annual cost of early stage 2 diabetes**<br>4x500mg metformin, 1x10mg ramipril 1x10mg amlodipine all daily, 4xGP appointments yearly, 5x nurse appointments yearly, 1 diet management programme every 4 years				314.33	5
**Total costs**					
Risk assessment				17.68	5,34,36,38
Annual monitoring cost, first year				102.51	
Annual monitoring cost, subsequent years				55.48	

eTable 9. Consolidated Health Economic Evaluation Reporting Standards (CHEERS) statement

Section/item	Item No	Recommendation	Reported on page, line number(s), figure, table
Title and abstract			
Title	1	Identify the study as an economic evaluation or use more specific terms such as "cost-effectiveness analysis", and describe the interventions compared.	Title Page, Lines 1-4
Abstract	2	Provide a structured summary of objectives, perspective, setting, methods (including study design and inputs), results (including base case and uncertainty analyses), and conclusions.	Pages 1-2
**Introduction**			
Background and objectives	3	Provide an explicit statement of the broader context for the study.	Page 3, Lines 4-23<br>Page 4, Lines 1-8
		Present the study question and its relevance for health policy or practice decisions.	Page 4, Lines 10-13
**Methods**			
Target population and subgroups	4	Describe characteristics of the base case population and subgroups analysed, including why they were chosen.	Pages 6-7<br>Page 10, Lines 21-23<br>Page 11<br>Page 12, Lines 1-2<br>Table 1<br>eTable 10<br>eFigures 3-5
Setting and location	5	State relevant aspects of the system(s) in which the decision(s) need(s) to be made.	Page 5, Lines 10-11<br>Page 12, Lines 5-7
Study perspective	6	Describe the perspective of the study and relate this to the costs being evaluated.	Page 13, Line 14-15
Comparators	7	Describe the interventions or strategies being compared and state why they were chosen.	Page 10, Lines 21-23<br>Pages 11-12<br>Page 13, Lines 1-6<br>Table 1<br>Table 2<br>eTable 10<br>eFigures 3-5
Time horizon	8	State the time horizon(s) over which costs and consequences are being evaluated and say why appropriate.	Page 13, Lines 9-10
Discount rate	9	Report the choice of discount rate(s) used for costs and outcomes and say why appropriate.	Page 13, Line 12

Section/item	Item No	Recommendation	Reported on page, line number(s), figure, table
Choice of health outcomes	10	Describe what outcomes were used as the measure(s) of benefit in the evaluation and their relevance for the type of analysis performed.	Page 8, Lines 4-6
Measurement of effectiveness	11a	*Single study-based estimates:* Describe fully the design features of the single effectiveness study and why the single study was a sufficient source of clinical effectiveness data.	Not applicable
	11b	*Synthesis-based estimates*: Describe fully the methods used for identification of included studies and synthesis of clinical effectiveness data.	Page 12, Lines 4-24<br>Page 13, Lines 1-6<br>Table 2<br>eTable 8
Measurement and valuation of preference based outcomes	12	If applicable, describe the population and methods used to elicit preferences for outcomes.	Page S6, Lines 32-42<br>Page S7, Lines 1-2<br>eTable 4
Estimating resources and costs	13a	*Single study-based economic evaluation:* Describe approaches used to estimate resource use associated with the alternative interventions. Describe primary or secondary research methods for valuing each resource item in terms of its unit cost. Describe any adjustments made to approximate to opportunity costs.	Not applicable
	13b	*Model-based economic evaluation:* Describe approaches and data sources used to estimate resource use associated with model health states. Describe primary or secondary research methods for valuing each resource item in terms of its unit cost. Describe any adjustments made to approximate to opportunity costs.	Page S7, Lines 4-28
Currency, price date, and conversion	14	Report the dates of the estimated resource quantities and unit costs. Describe methods for adjusting estimated unit costs to the year of reported costs if necessary. Describe methods for converting costs into a common currency base and the exchange rate.	Page 13, Lines 9-14
Choice of model	15	Describe and give reasons for the specific type of decision-analytical model used. Providing a figure to show model structure is strongly recommended.	Page 8<br>Page 9, Lines 1-17<br>Page S4, Lines 3-10

Section/item	Item No	Recommendation	Reported on page, line number(s), figure, table
Assumptions	16	Describe all structural or other assumptions underpinning the decision-analytical model.	Page 8, Lines 13-24<br>Page 9, Lines 1-17<br>Page S4, Lines 12-26<br>eTables 1-6
Analytical methods	17	Describe all analytical methods supporting the evaluation. This could include methods for dealing with skewed, missing, or censored data; extrapolation methods; methods for pooling data; approaches to validate or make adjustments (such as half cycle corrections) to a model; and methods for handling population heterogeneity and uncertainty.	Page 9, Lines 19-23<br>Page 10, Lines 1-19<br>Page 11, Lines 10-23<br>Pages 12-14<br>Page 15, Lines 1-20<br>Pages S4-S8
**Results**			
Study parameters	18	Report the values, ranges, references, and, if used, probability distributions for all parameters. Report reasons or sources for distributions used to represent uncertainty where appropriate. Providing a table to show the input values is strongly recommended.	Page 15, Lines 23-24<br>Page 11, Lines 1-14<br>Table 1<br>Table 2<br>eTable 8<br>eTable 10
Incremental costs and outcomes	19	For each intervention, report mean values for the main categories of estimated costs and outcomes of interest, as well as mean differences between the comparator groups. If applicable, report incremental cost-effectiveness ratios.	Page 16, Lines 6-23<br>Page 17, Lines 1-13<br>Table 3<br>Figure 2<br>eTables 11-12
Characterising uncertainty	20a	*Single study-based economic evaluation:* Describe the effects of sampling uncertainty for the estimated incremental cost and incremental effectiveness parameters, together with the impact of methodological assumptions (such as discount rate, study perspective).	n/a
	20b	*Model-based economic evaluation:* Describe the effects on the results of uncertainty for all input parameters, and uncertainty related to the structure of the model and assumptions.	Page 17, Lines 13-21<br>Figure 4<br>eFigures 6-8<br>eTable 13-14
Characterising heterogeneity	21	If applicable, report differences in costs, outcomes, or cost-effectiveness that can be explained by variations between subgroups of patients with different baseline characteristics or other observed variability	Page 17, Lines 13-21<br>eTable 13-14

Section/item	Item No	Recommendation	Reported on page, line number(s), figure, table
		in effects that are not reducible by more information.	
**Discussion**			
Study findings, limitations, generalisability, and current knowledge	22	Summarise key study findings and describe how they support the conclusions reached. Discuss limitations and the generalisability of the findings and how the findings fit with current knowledge.	Pages 18-19<br>Page 20, Lines 1-8
**Other**			
Source of funding	23	Describe how the study was funded and the role of the funder in the identification, design, conduct, and reporting of the analysis. Describe other non-monetary sources of support.	Page 21, Lines 7-15
Conflicts of interest	24	Describe any potential for conflict of interest of study contributors in accordance with journal policy. In the absence of a journal policy, we recommend authors comply with International Committee of Medical Journal Editors recommendations.	Page 21, Lines 1-5

eTable 10: Descriptive statistics of patient populations

		Overall	ASSIGN 20	ARR 20	Age-Strat 20	ASSIGN 10	ARR 10	Age-Strat 10
Average Value	**Male (%)**	42.4	44.9	46.0	56.4	46.6	46.3	52.0
	**Diabetes (%)**	6.8	16.4	14.8	18.9	11.3	10.7	11.6
	**Family History (%)**	45.3	65.1	63.4	61.2	61.4	59.3	57.1
	**Smoker (%)**	19.1	25.5	25.2	32.5	23.2	22.7	26.5
	**Age**	58.9	71.9	70.4	59.4	66.2	65.5	59.2
	**SIMD**	19.4	20.7	20.5	22.4	20.1	19.9	21.3
	**CPD**	3.0	4.4	4.3	5.9	3.8	3.7	4.5
	**SBP (mmHg)**	130.6	134.4	134.3	138.0	133.1	133.1	135.5
	**TC (mmol/L)**	5.60	5.78	6.05	5.92	5.71	5.86	5.78
	**HDL-C (mmol/L)**	1.52	1.40	1.37	1.30	1.45	1.43	1.38
	**Non-HDL-C (mmol/L)**	4.07	4.38	4.68	4.62	4.26	4.43	4.40

eTable 11: Cost-effectiveness results, multiple ASSIGN score thresholds

ASSIGN Score Threshold	Number Treated	Mean Discounted QALYs Gained	Mean Discounted Cost (£1000's)	ICER (£/QALY)
**No Treatment**	0	Reference		
**20%**	803,937	103,302	397,290,926	3,846
**19%**	836,671	106,822	428,803,527	8,952
**18%**	880,630	111,699	472,497,130	8,958
**17%**	928,640	117,294	525,563,559	9,486
**16%**	984,318	123,792	590,276,914	9,958
**15%**	1,055,021	131,557	675,555,930	10,983
**14%**	1,118,110	138,336	756,677,372	11,966
**13%**	1,185,220	145,531	847,551,292	12,630
**12%**	1,267,716	154,069	963,903,281	13,629
**11%**	1,353,286	163,014	1,095,526,384	14,715
**10%**	1,445,741	172,352	1,243,768,601	15,874
**9%**	1,554,326	182,693	1,423,577,360	17,387
**8%**	1,657,364	191,971	1,604,581,196	19,510
**7%**	1,768,072	201,475	1,810,704,786	21,687
**6%**	1,902,167	212,121	2,070,679,066	24,422
**5%**	2,042,977	222,797	2,361,062,843	27,199
**4%**	2,189,865	232,599	2,675,498,770	32,079
**3%**	2,341,711	241,270	3,011,579,250	38,760
**2%**	2,451,868	246,097	3,258,325,600	51,121
**1%**	2,506,688	247,589	3,378,759,366	80,697
**All Treated**	2,507,179	247,361	3,347,207,271	138,531

eTable 12: Disaggregated cost of different policies

Policy	Discounted Costs (£1000's), Mean from Probabilistic Analysis					
	CVD	Non-CVD	Statin	Monitoring	Risk Assessment	Total
No Treatment	*Reference*					
ASSIGN 20	-1,074,107	892,725	91,858	438,949	46,163	397,291
ARR 20	-1,195,072	1,047,826	97,202	461,698	46,163	459,537
Age-Strat 20	-1,330,487	1,380,740	126,787	587,228	46,163	812,586
ASSIGN 10	-1,943,708	1,973,038	204,969	959,317	46,163	1,243,769
ARR 10	-2,044,253	2,143,191	209,498	978,572	46,163	1,337,161
Age-Strat 10	-2,130,339	2,419,345	240,943	1,112,010	46,163	1,692,575

eTable 13: Cost-effectiveness results, scenario analysis – reduced statin therapy adherence

Policy	Number Treated	Primary CVD Events Prevented	Undiscounted Life Years	Discounted QALYs Gained	Discounted Cost (£1000's)	ICER (£/QALY)
**Expanding treatment eligibility**						
**No Treatment**	0	*Reference*				
**ASSIGN 20**	804,000	17,600 (10,800-25,300)	122,000 (56,000-206,000)	53,000 (25,200-86,000)	219,000 (41,900-421,000)	4,120 (1,200-10,700)
**ASSIGN 10**	1,445,500	31,500 (19,500-45,200)	235,000 (103,000-396,000)	87,900 (40,000-144,000)	646,000 (315,000-1,030,000)	12,300 (7,670-26,700)
**Policies comparable to ASSIGN 20**						
**ASSIGN 20**	804,000	*Reference*				
**ARR 20**	804,000	1,360 (724-2,190)	13,200 (2,260-26,100)	4,400 (1,780-7,630)	30,700 (11,620-54,500)	6,990 (4,360-11,200)
**Age-Strat 20**	804,000	746 (-302-1,930)	11,500 (-43,000-56,500)	-3,170 (-11,900-4,490)	211,000 (116,000-310,000)	Dominated* (Dominated-Dominated)
**Policies comparable to ASSIGN 10**						
**ASSIGN 10**	1,445,500	*Reference*				
**ARR 10**	1,445,500	1,370 (740-2,240)	15,900 (4,070-30,800)	3,970 (1,810-6,700)	46,800 (22,900-77,300)	11,700 (8,590-17,500)
**Age-Strat 10**	1,445,500	1,150 (205-2,360)	16,700 (-32,800-60,500)	-3,950 (-11,400-2,720)	227,000 (125,000-335,000)	Dominated* (Dominated-Dominated)

eTable 14: Cost-effectiveness results, scenario analysis – individuals with diabetes removed from prospective patient population

Policy	Number Treated	Primary CVD Events Prevented	Undiscounted Life Years	Discounted QALYs Gained	Discounted Cost (£1000's)	ICER (£/QALY)
**Expanding treatment eligibility**						
**No Treatment**	0	*Reference*				
**ASSIGN 20**	672,000	30,300 (18,800-43,500)	122,000 (63,400-195,600)	89,500 (42,600-145,000)	372,000 (71,900-718,000)	4,150 (1,270-10,200)
**ASSIGN 10**	1,283,000	57,000 (35,700-81,700)	217,000 (114,000-348,000)	156,000 (69,300-257,000)	1,177,000 (580,000-1,895,000)	12,100 (7,630-26,000)
**Policies comparable to ASSIGN 20**						
**ASSIGN 20**	672,000	*Reference*				
**ARR 20**	685,000	2,950 (1,880-4,330)	11,000 (4,930-18,200)	9,300 (4,040-15,300)	65,200 (29,800-109,000)	7,020 (4,500-11,000)
**Age-Strat 20**	652,000	1,050 (197-2,180)	-12,900 (-35,200-6,780)	-7,060 (-22,200-7,340)	380,000 (207,000-579,000)	Dominated* (Dominated-Dominated)
**Policies comparable to ASSIGN 10**						
**ASSIGN 10**	1,283,000	*Reference*				
**ARR 10**	1,291,000	2,920 (1,880-4,250)	9,918 (4,400-16,500)	8,240 (3,580-13,500)	101,000 (60,700-150,000)	12,300 (9,520-19,000)
**Age-Strat 10**	1,277,000	2,380 (1,270-3,930)	-10,700 (-28,300-5,910)	-7,410 (-21,400-5,240)	438,000 (261,000-644,000)	Dominated* (Dominated-Dominated)

Supplemental Figures

eFigure 1: Predicted versus observed cumulative incidence of primary events in placebo arm of the WOSCOPS trial, from Lewsey et al.[19]

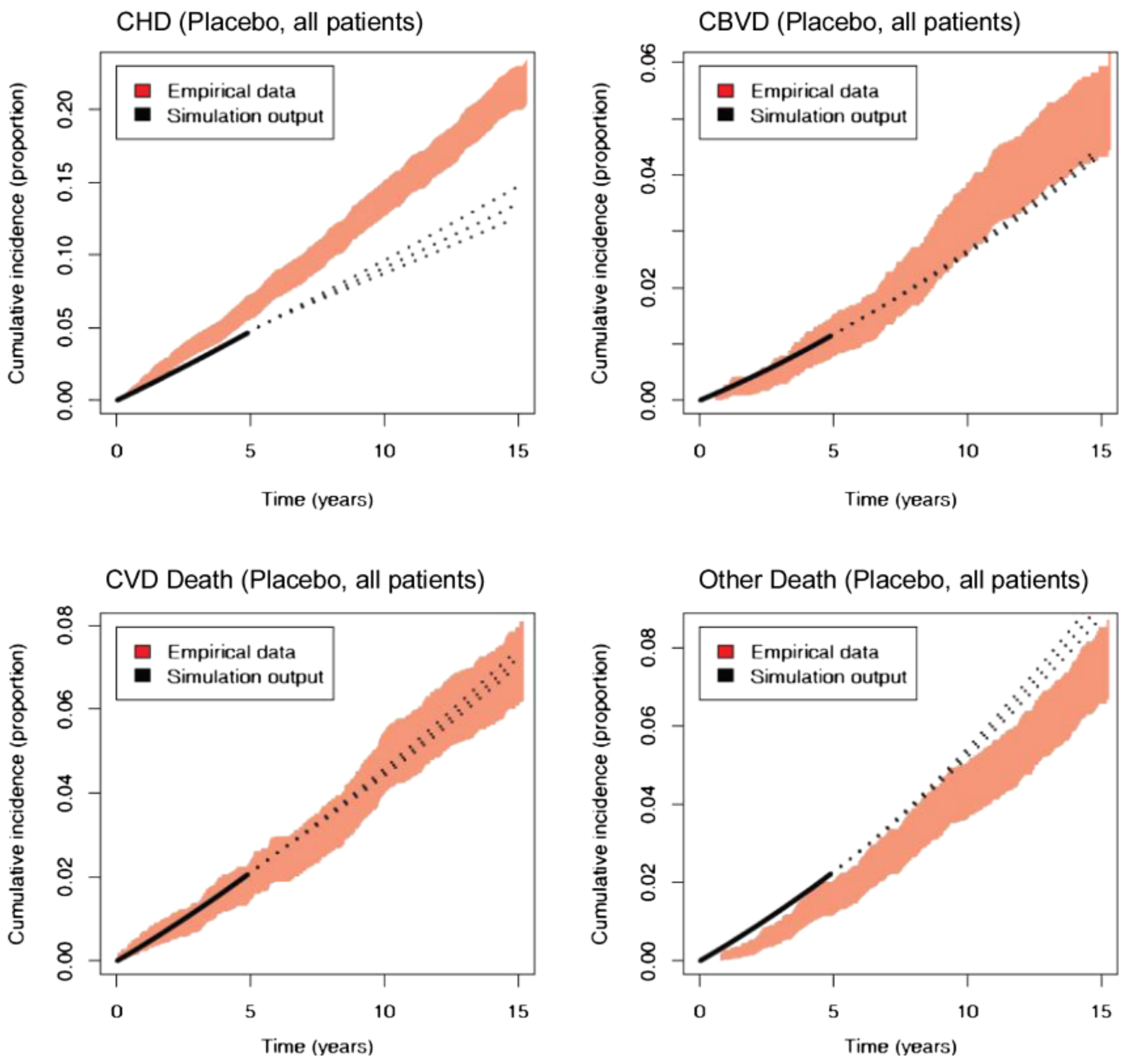

eFigure 2: Predicted versus observed cumulative incidence of primary events in treatment arm of the WOSCOPS trial, from Lewsey et al.[19]

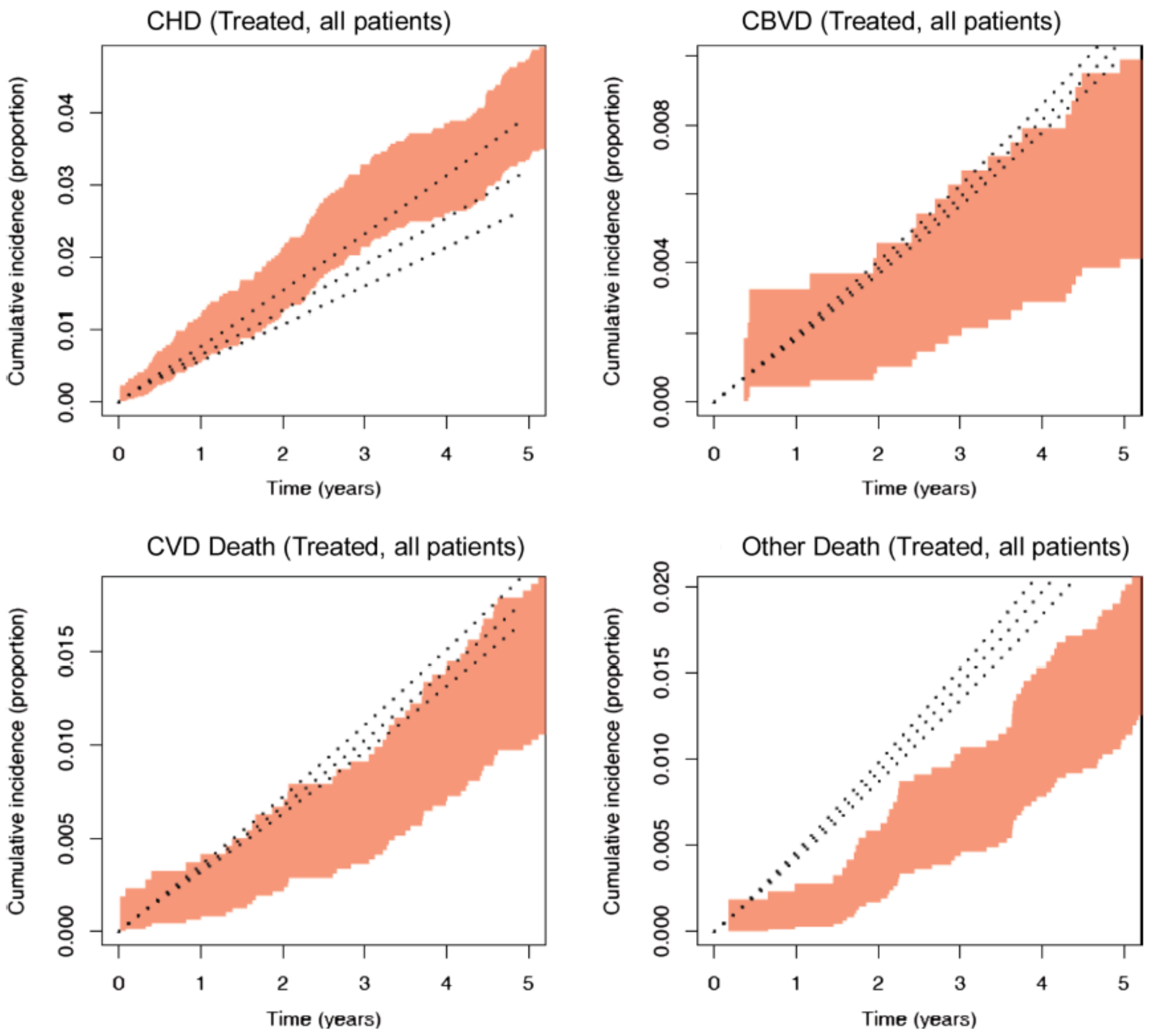

eFigure 3: Proportion of population eligible for treatment with different risk thresholds

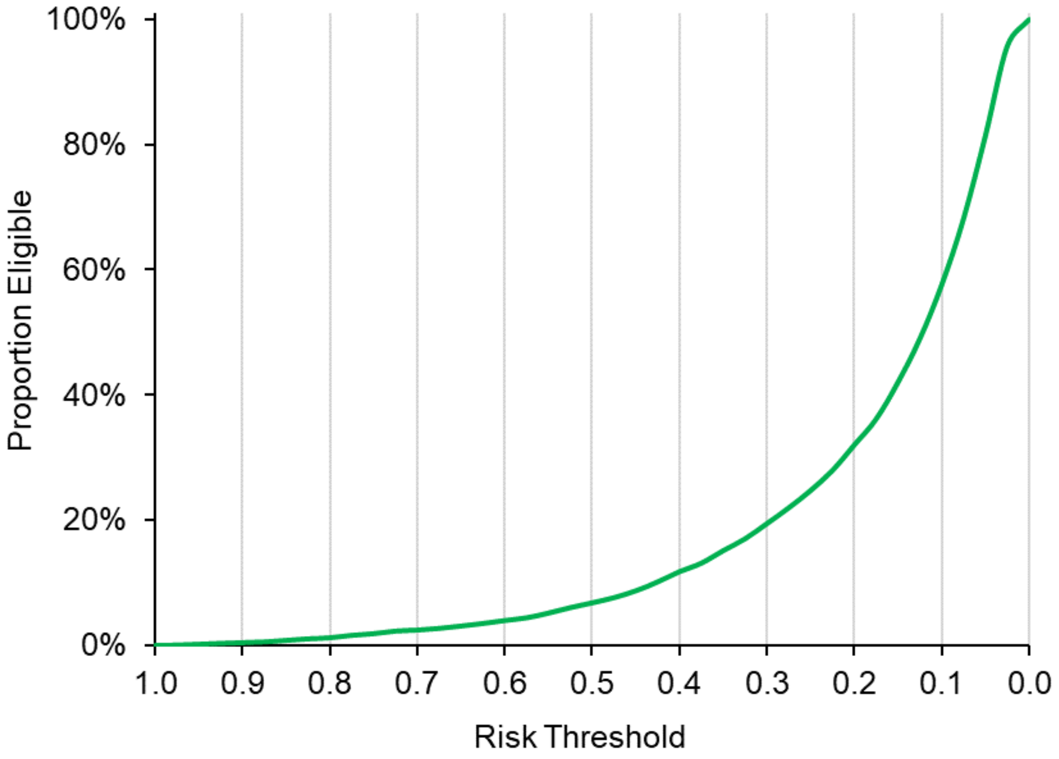

eFigure 4: Proportion of age-groups eligible for treatment with different risk thresholds

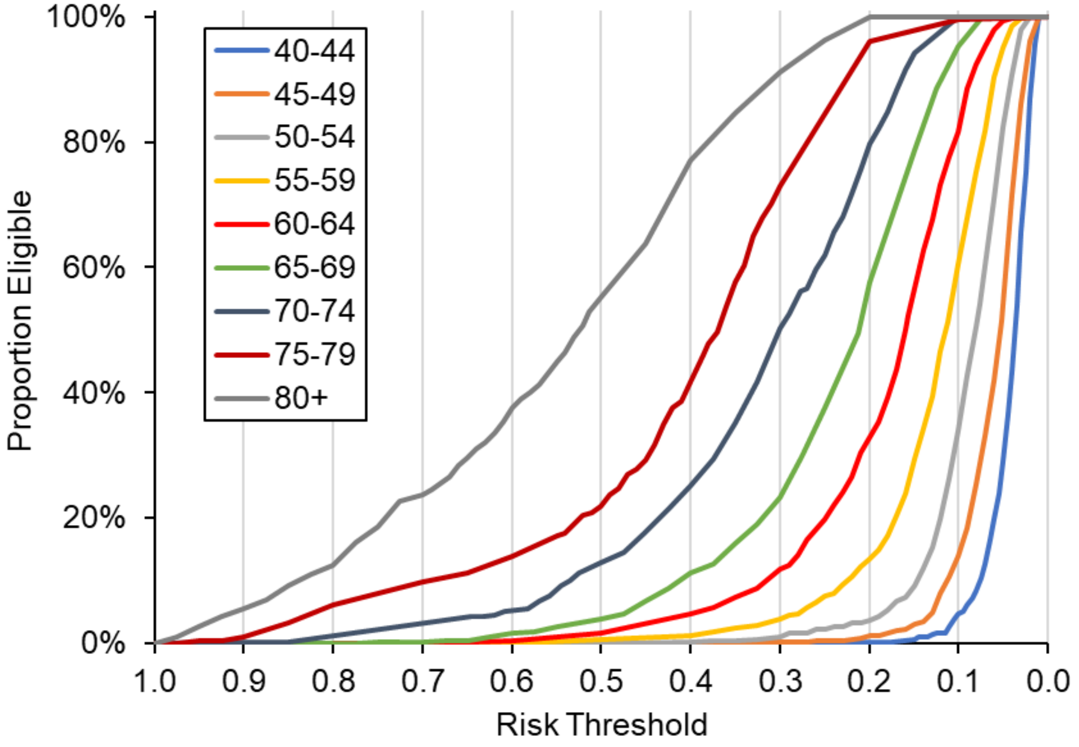

eFigure 5: Proportion of population eligible for treatment with different absolute risk reduction thresholds

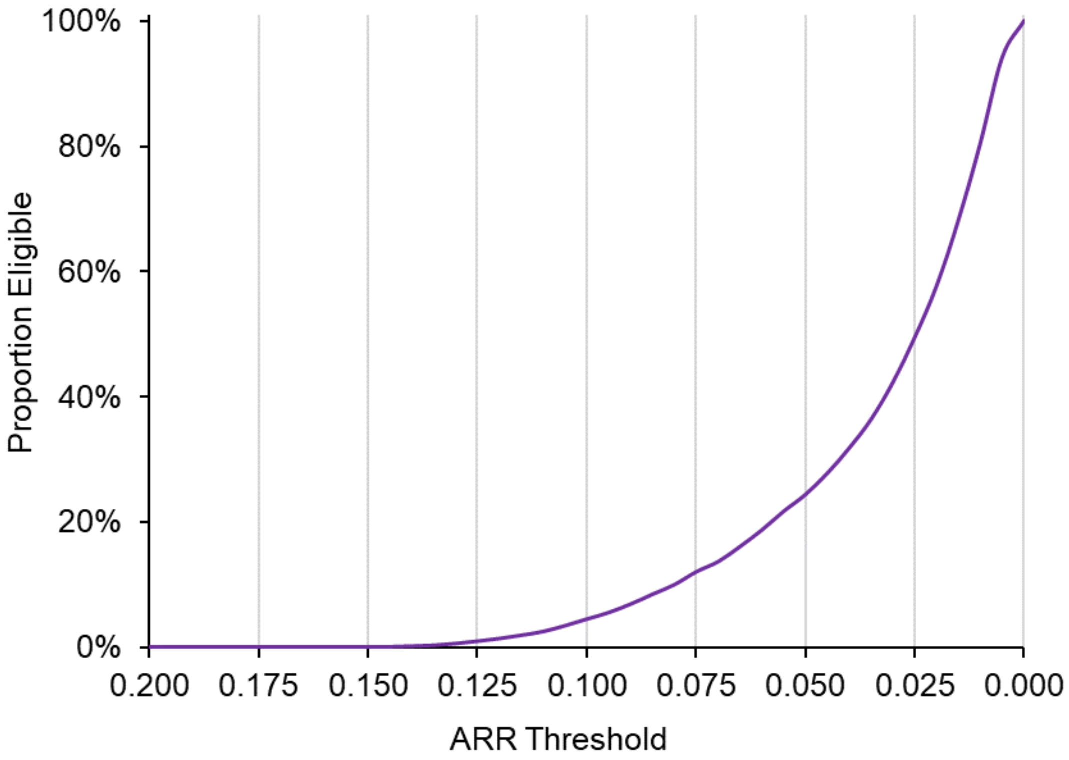

eFigure 6A: Tornado diagrams for strategies treating same number as ASSIGN 20
QALYs valued at £20,000. Increased net monetary benefit indicates increased cost-effectiveness

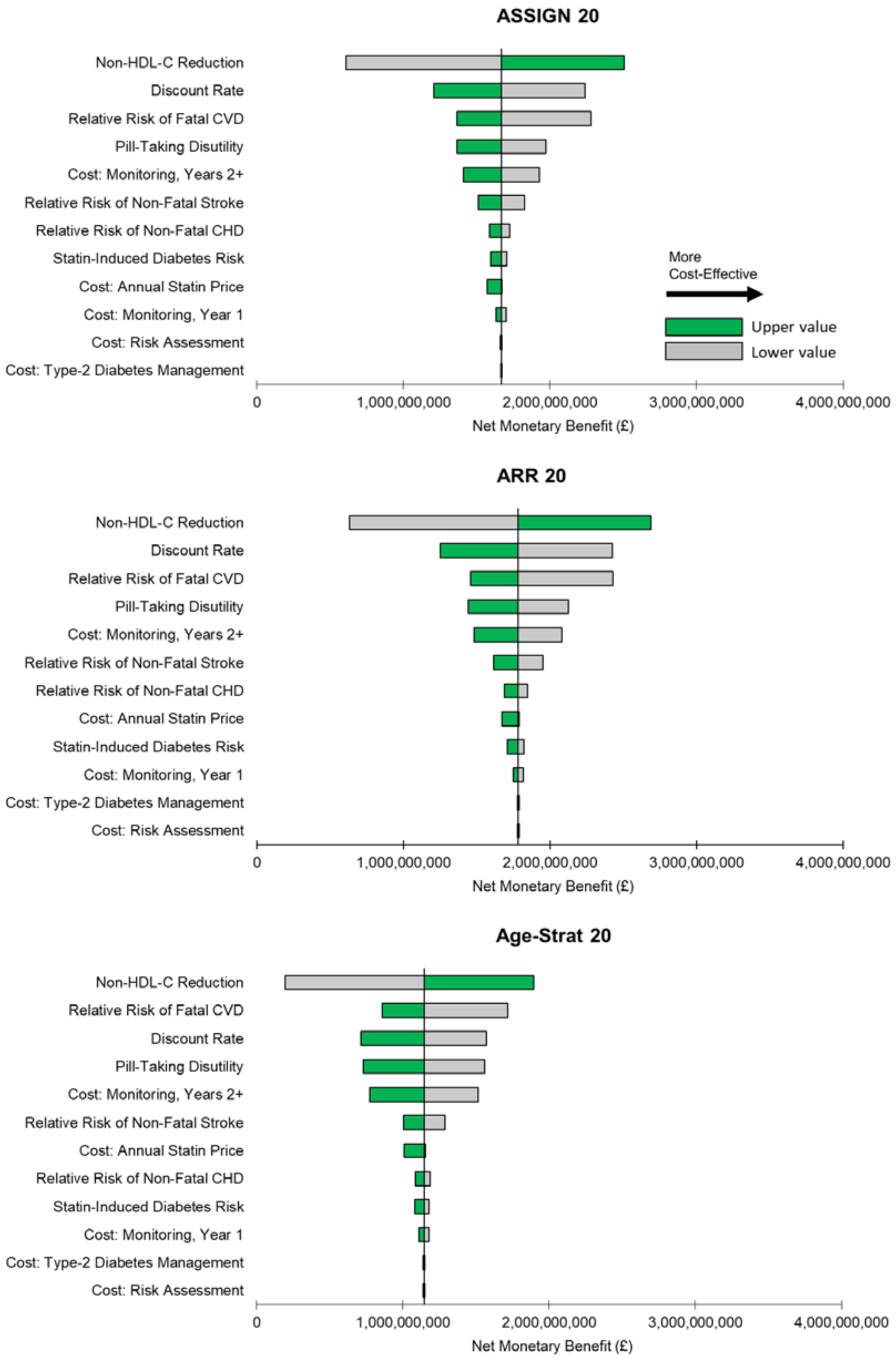

eFigure 6B: Tornado diagrams for strategies treating same number as ASSIGN 10
QALYs valued at £20,000. Increased net monetary benefit indicates increased cost-effectiveness

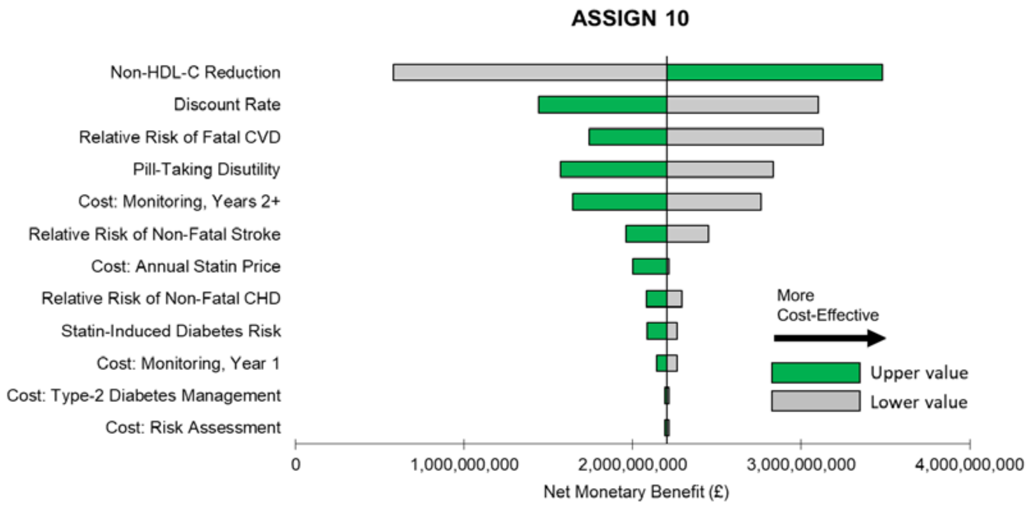

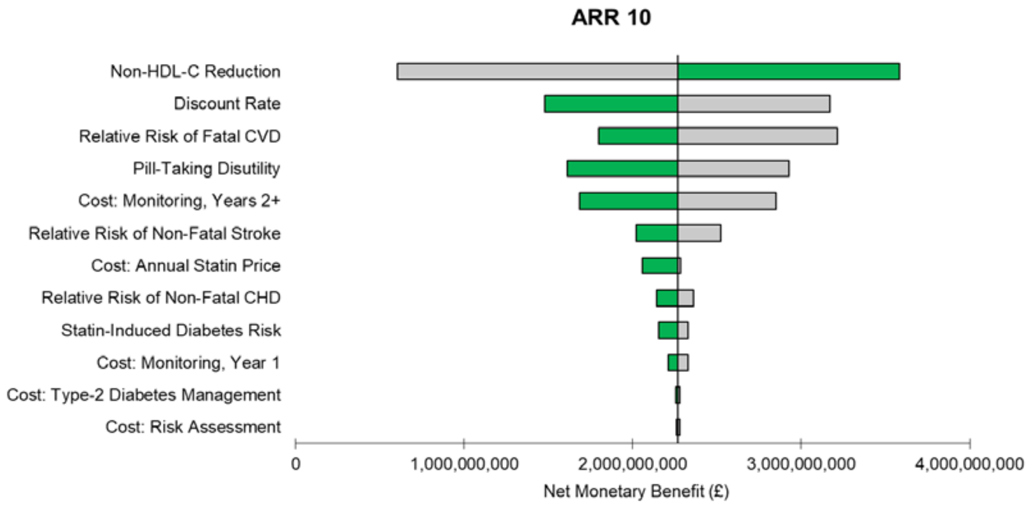

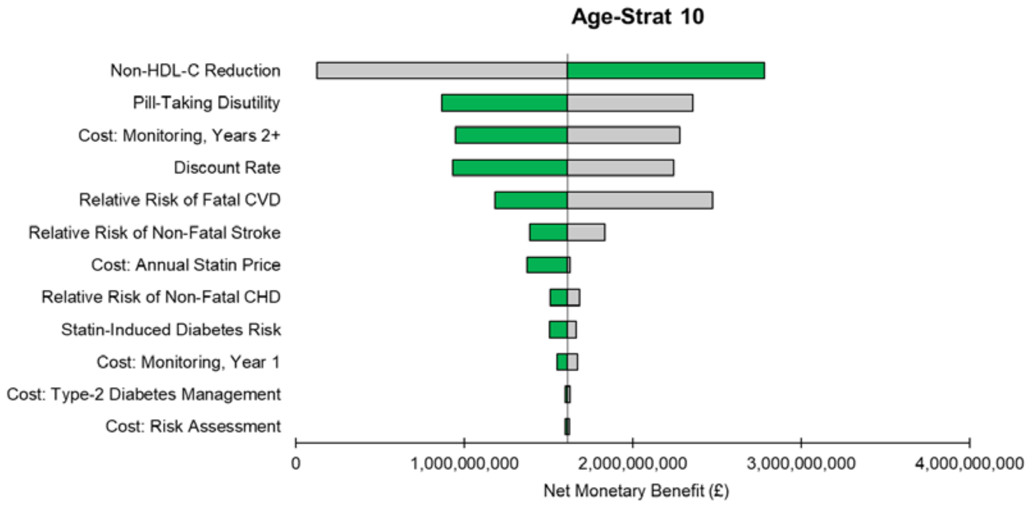

eFigure 7: Graphical representation of one-way sensitivity analysis results

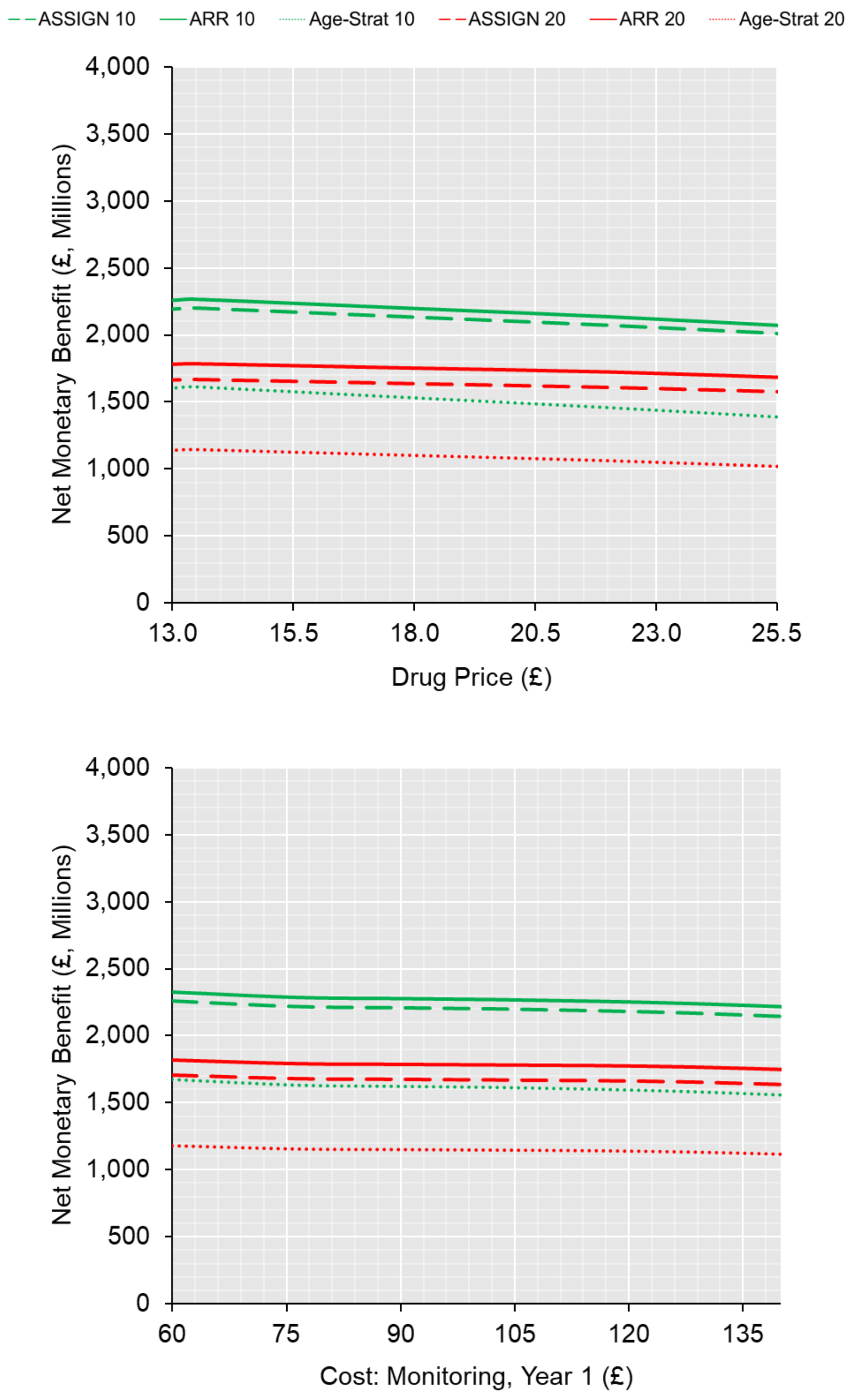

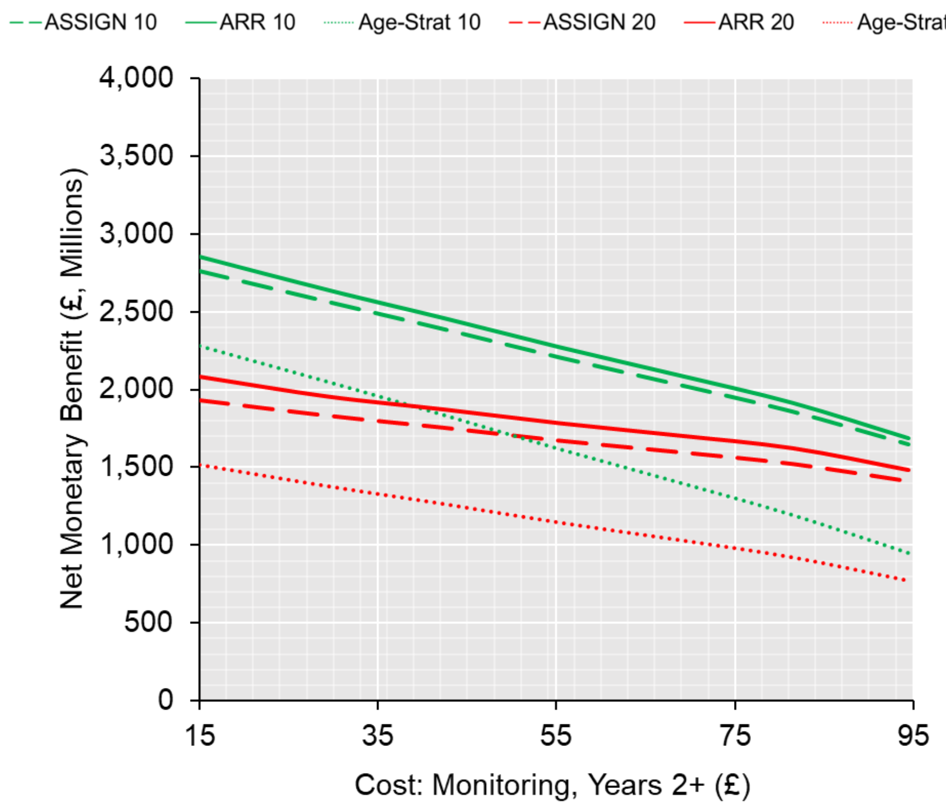
ASSIGN 10
ARR 10
Age-Strat 10
ASSIGN 20
ARR 20
Age-Strat 20
4,000
3,500
3,000
2,500
2,000
1,500
1,000
500
0
Net Monetary Benefit (£, Millions)
15
35
55
75
95
Cost: Monitoring, Years 2+ (£)

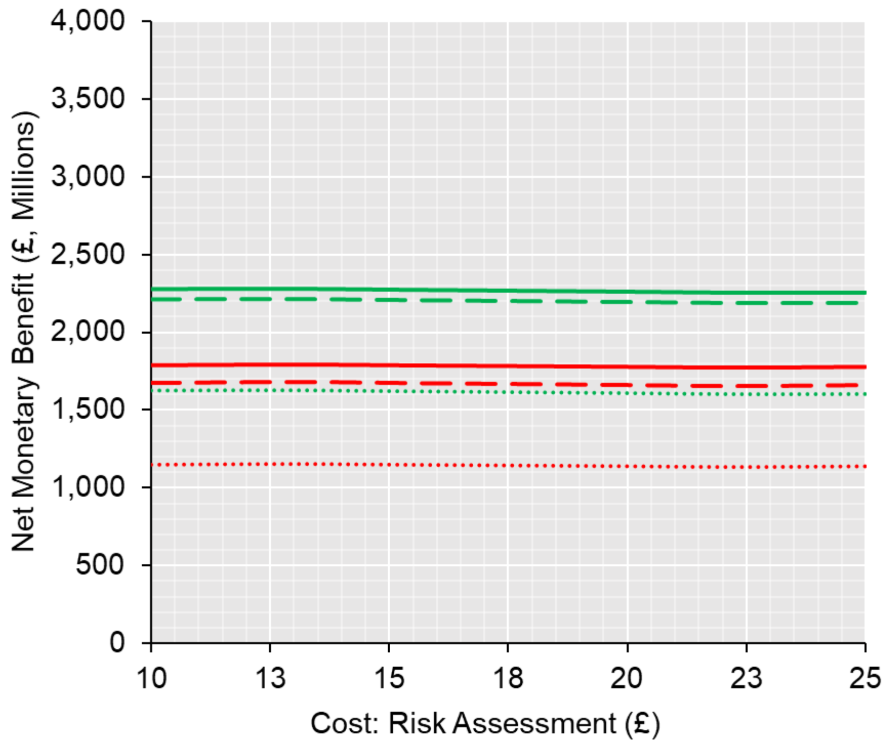
4,000
3,500
3,000
2,500
2,000
1,500
1,000
500
0
Net Monetary Benefit (£, Millions)
10
13
15
18
20
23
25
Cost: Risk Assessment (£)

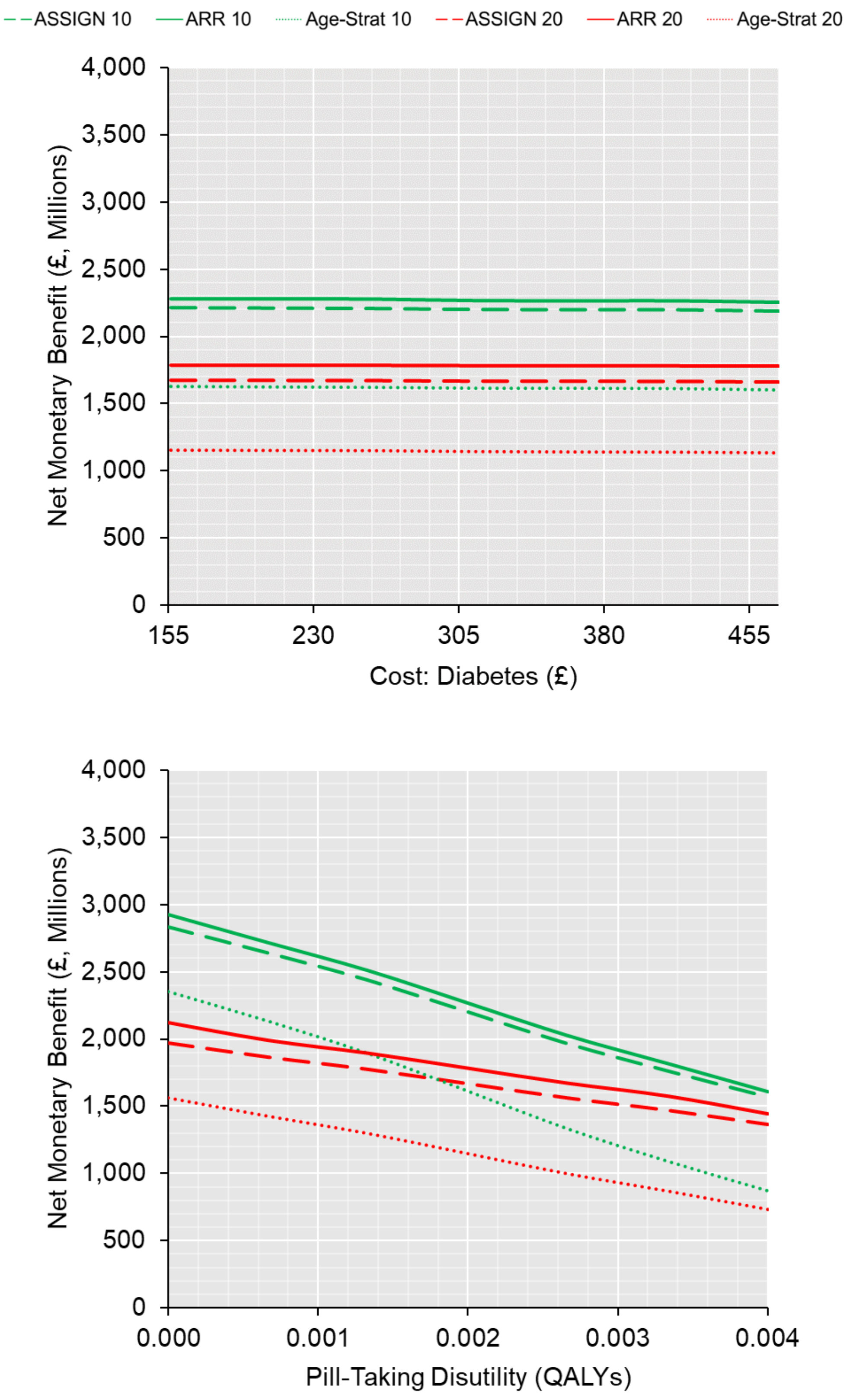
ASSIGN 10
ARR 10
Age-Strat 10
ASSIGN 20
ARR 20
Age-Strat 20
Net Monetary Benefit (£, Millions)
4,000
3,500
3,000
2,500
2,000
1,500
1,000
500
0
155
230
305
380
455
Cost: Diabetes (£)
Net Monetary Benefit (£, Millions)
4,000
3,500
3,000
2,500
2,000
1,500
1,000
500
0
0.000
0.001
0.002
0.003
0.004
Pill-Taking Disutility (QALYs)

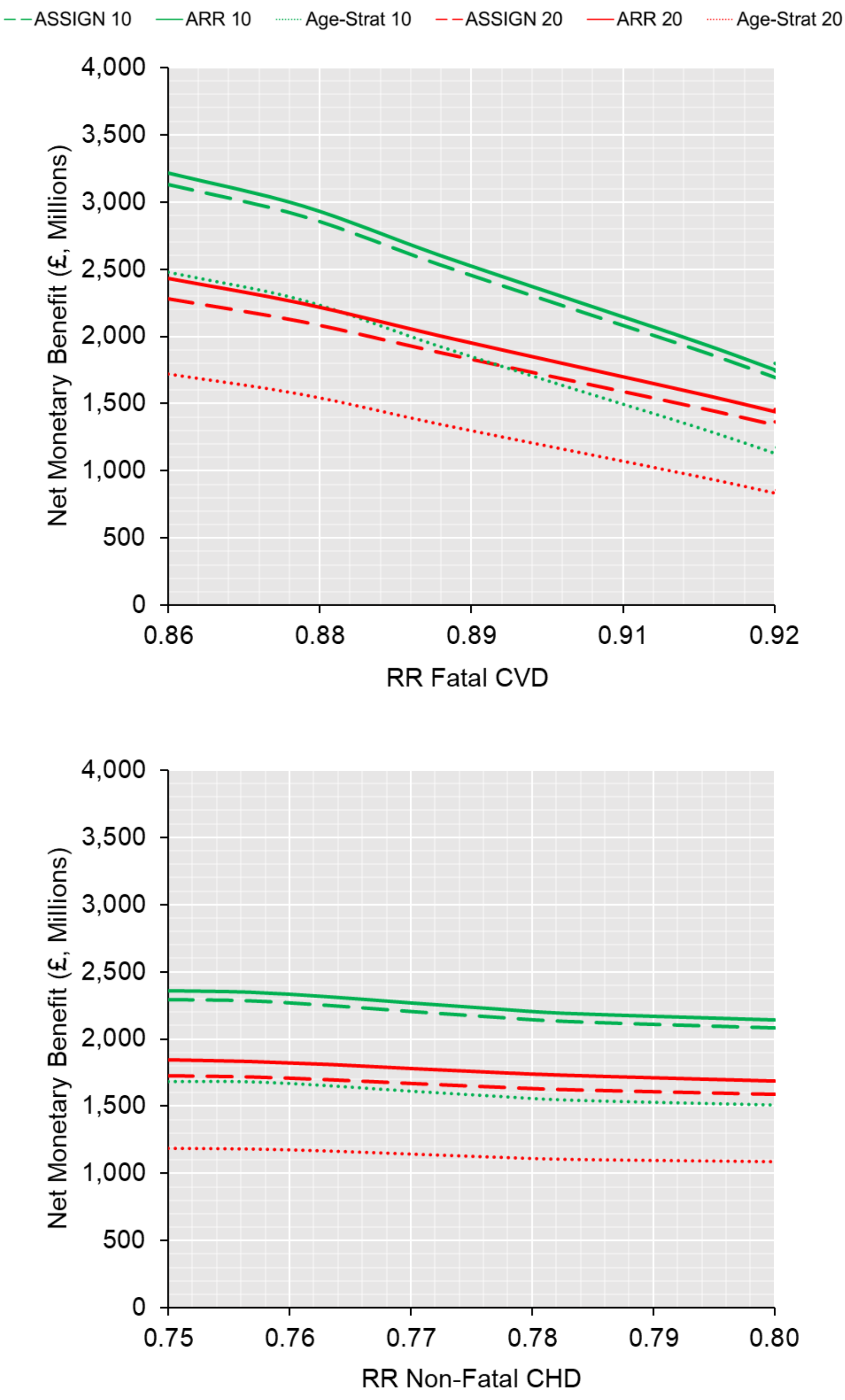
ASSIGN 10
ARR 10
Age-Strat 10
ASSIGN 20
ARR 20
Age-Strat 20
Net Monetary Benefit (£, Millions)
4,000
3,500
3,000
2,500
2,000
1,500
1,000
500
0
0.86
0.88
0.89
0.91
0.92
RR Fatal CVD
Net Monetary Benefit (£, Millions)
4,000
3,500
3,000
2,500
2,000
1,500
1,000
500
0
0.75
0.76
0.77
0.78
0.79
0.80
RR Non-Fatal CHD

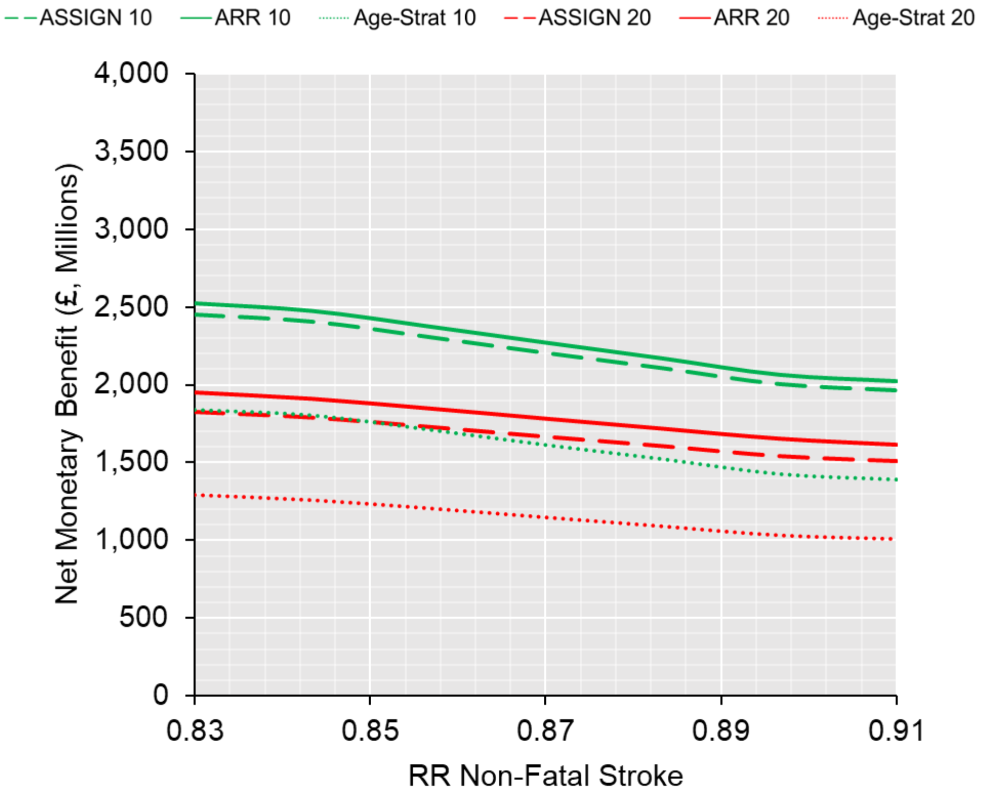
ASSIGN 10
ARR 10
Age-Strat 10
ASSIGN 20
ARR 20
Age-Strat 20
Net Monetary Benefit (£, Millions)
4,000
3,500
3,000
2,500
2,000
1,500
1,000
500
0
0.83
0.85
0.87
0.89
0.91
RR Non-Fatal Stroke

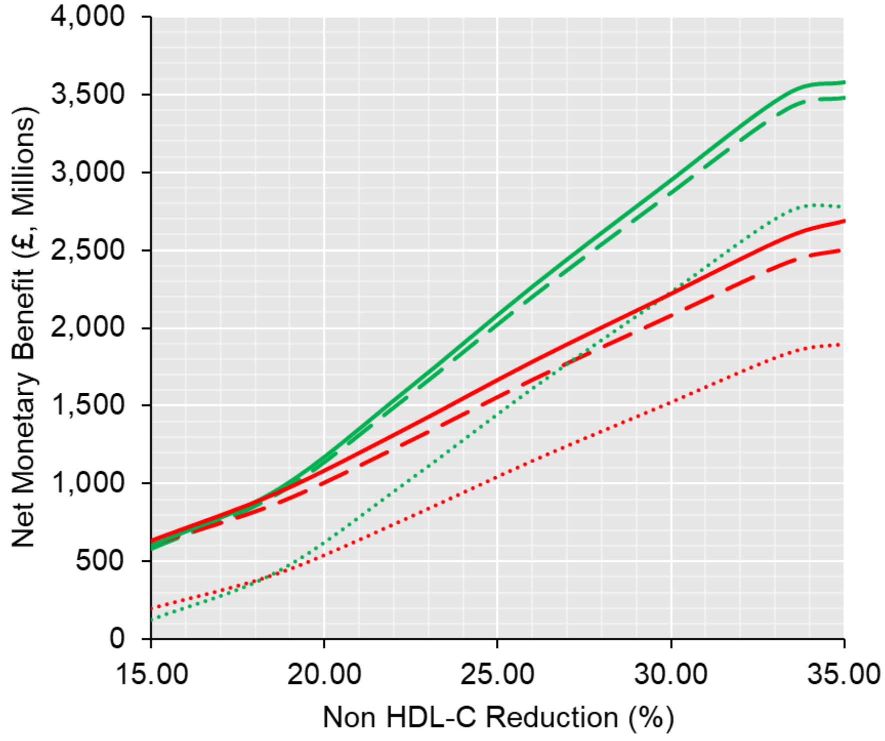
Net Monetary Benefit (£, Millions)
4,000
3,500
3,000
2,500
2,000
1,500
1,000
500
0
15.00
20.00
25.00
30.00
35.00
Non HDL-C Reduction (%)

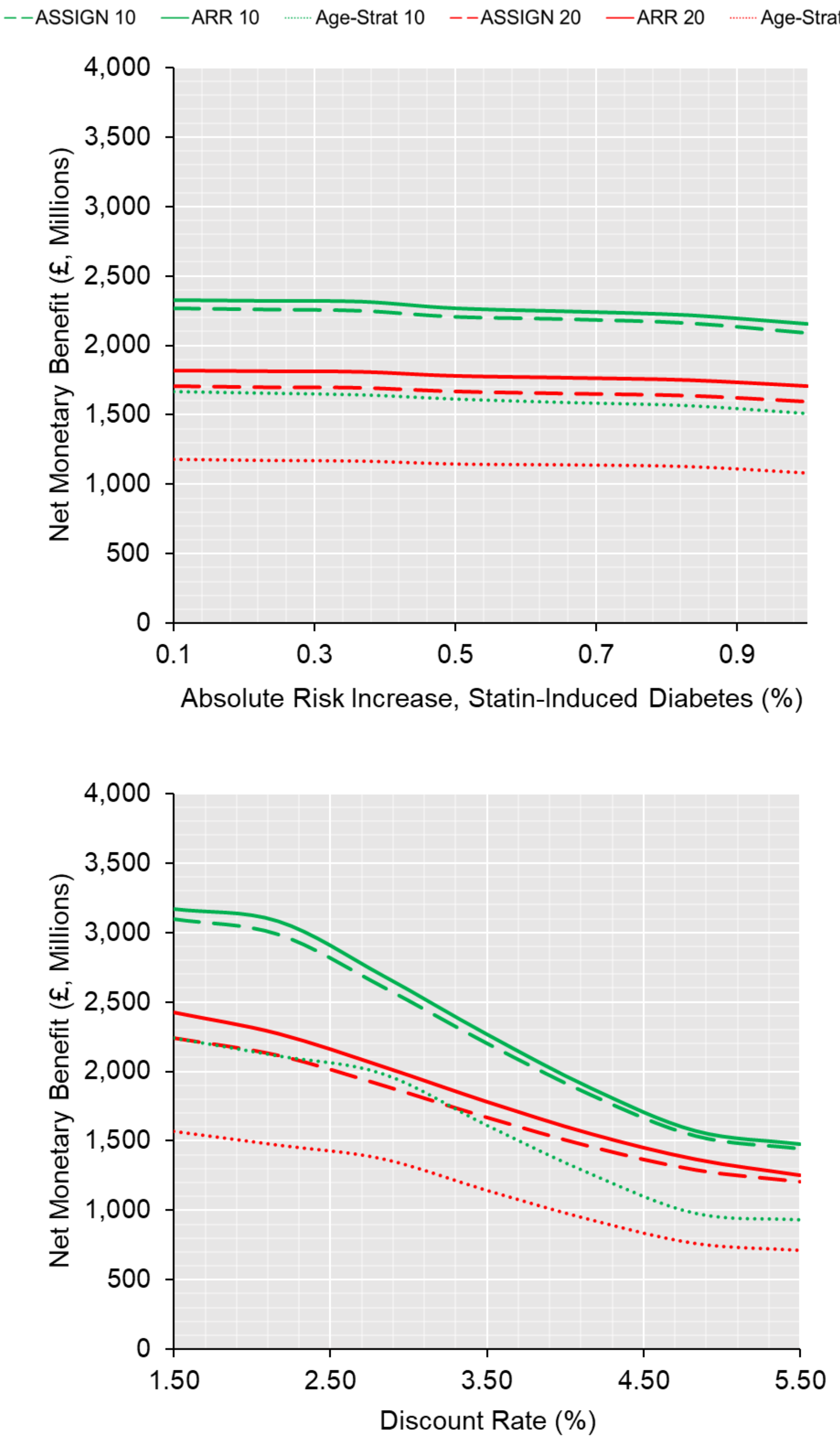
ASSIGN 10
ARR 10
Age-Strat 10
ASSIGN 20
ARR 20
Age-Strat 20
Net Monetary Benefit (£, Millions)
4,000
3,500
3,000
2,500
2,000
1,500
1,000
500
0
0.1
0.3
0.5
0.7
0.9
Absolute Risk Increase, Statin-Induced Diabetes (%)
Net Monetary Benefit (£, Millions)
4,000
3,500
3,000
2,500
2,000
1,500
1,000
500
0
1.50
2.50
3.50
4.50
5.50
Discount Rate (%)

eFigure 8: Net monetary benefit of treatment strategies versus pill-taking disutility

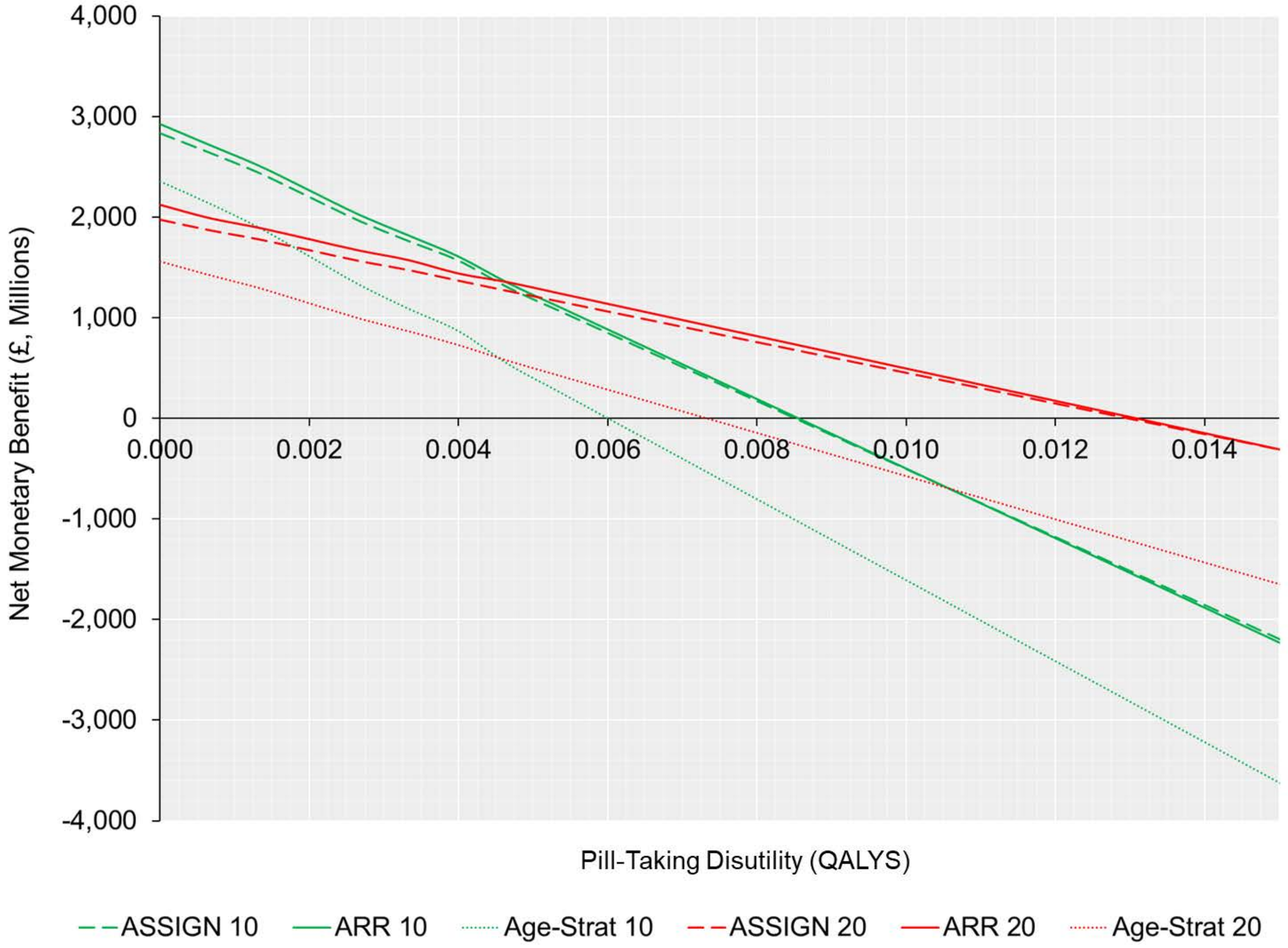

Supplement: Supplementary file 1 [file cir-145-1312-s001.pdf]
